# Substrate Specificity of the Flavoenzyme BhaC_1_ That Converts a C-Terminal Trp to a Hydroxyquinone

**DOI:** 10.1021/acs.biochem.2c00206

**Published:** 2022-05-25

**Authors:** Page N. Daniels, Wilfred A. van der Donk

**Affiliations:** † Department of Biochemistry, University of Illinois at Urbana-Champaign, Urbana, Illinois 61801, United States; ‡ Department of Chemistry and Howard Hughes Medical Institute, University of Illinois at Urbana-Champaign, Urbana, Illinois 61801, United States; § Carl R. Woese Institute for Genomic Biology, University of Illinois at Urbana-Champaign, Urbana, Illinois 61801, United States

## Abstract

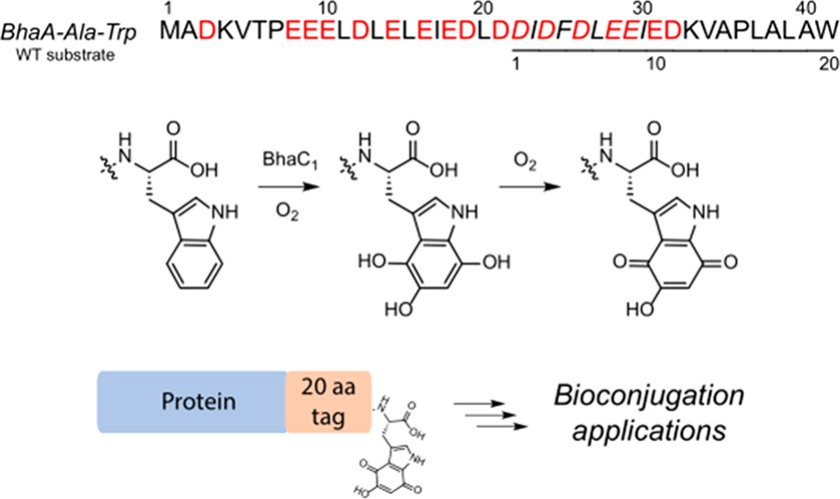

The preparation of protein–protein, protein–peptide, and protein–small molecule conjugates is important for a variety of applications, such as vaccine production, immunotherapies, preparation of antibody–drug conjugates, and targeted delivery of therapeutics. To achieve site-selective conjugation, selective chemical or enzymatic functionalization of proteins is required. We have recently reported biosynthetic pathways in which small, catalytic scaffold peptides are utilized for the generation of amino acid-derived natural products called pearlins. In these systems, peptide amino-acyl tRNA ligases (PEARLs) append amino acids to the C-terminus of a scaffold peptide, and tailoring enzymes encoded in the biosynthetic gene clusters modify the PEARL-appended amino acid to generate a variety of natural products. Herein, we investigate the substrate selectivity of one such tailoring enzyme, BhaC_1_, that participates in pyrroloiminoquinone biosynthesis. BhaC_1_ converts the indole of a C-terminal tryptophan into an *o*-hydroxy-*p*-quinone, a promising moiety for site-selective bioconjugation. Our studies demonstrate that BhaC_1_ requires a 20-amino acid peptide for substrate recognition. When this peptide was appended at the C-terminus of proteins, the C-terminal Trp was modified by BhaC_1_. The enzyme is sufficiently selective that only small changes to the sequence of the peptide are tolerated. An AlphaFold model for substrate recognition explains the selectivity of the enzyme, which may be used to install a reactive handle onto the C-terminus of proteins.

Site-selective post-translational or chemical modification of proteins has long been a goal of chemical biology and biomolecular engineering research.^1−3^ The ability to site-specifically modify a protein allows selective installation of therapeutic small molecules, fluorophores, and mimics of post-translational modifications, which in turn has advanced our understanding of biology and has facilitated development of new drug modalities.^4−7^ The current strategies for bioconjugation include introduction of unnatural amino acids into the backbone sequence using stop-codon suppression strategies,^8−10^ site-selective modification of cysteine or lysine residues,^11,12^ ligation-based strategies,^13−18^ chemoselective reactions on the N-terminus^19−22^ or the side chains of specific amino acids,^23−27^ and enzymatic introduction of reactive handles.^4,28−34^ Despite the advances in selective modification of proteins, many strategies for labeling lead to heterogeneous mixtures and can result in low yields. Furthermore, for decoration of proteins with multiple conjugates, additional complementary methods will be valuable.

The coupling of proteins with native functional groups can be achieved through ligations or sortase-mediated reactions, but typically only at the N- and C-termini.^17,35^ The N-terminus of proteins has been used extensively for selective modifications,^19−22,36,37^ but the C-terminus of proteins also provides a putative site for selective modification. However, the C-terminus has historically been challenging to functionalize.^18,35,37^ The utilization of enzymes to install functional handles on biomolecules for bioconjugation could mitigate some of the issues encountered with chemical methods because enzymes typically require mild reaction conditions, are often site-selective, and can in principle also be used in cells. Herein, we report the substrate specificity of an enzyme that site-selectively modifies a C-terminal Trp to an electrophilic handle for subsequent chemistry.

During our studies on PEARL-based biosynthesis of pyrroloiminoquinone-type natural products, we discovered a novel tetratricopeptide (TPR) domain-containing, flavin mononucleotide (FMN)-dependent enzyme class that trihydroxylates the indole of a C-terminal tryptophan residue (Figure 1A,B).^38^ In the biosynthesis of the pyrroloiminoquinone core scaffold, the trihydroxylated product of BhaC_1_ oxidizes to an *o*-hydroxy-*p*-quinone (Figure 1B), which is then the substrate for further structural elaboration to generate natural products like ammosamide. The oxidation catalyzed by BhaC_1_ is a rare example of post-translational trihydroxylation by a single enzyme. On the basis of previous studies, the reactive *o*-hydroxy-*p*-quinone generated by BhaC_1_ will be a candidate for site-selective bioconjugation.^30^ Quinones are Michael-type acceptors and readily react with nucleophiles like sulfhydryl groups. *p*-Quinones react via 1,4-reductive addition reactions, which generate the hydroquinone with covalent attachment. Reactions with nitrogen nucleophiles such as histidine, lysine, N-terminal amino acids, and purine and pyrimidine bases on DNA are slower than sulfur-based nucleophilic additions.^39−41^*o-*Hydroxy-*p-*quinones undergo substitution reactions by addition–elimination processes. Amino acid-derived quinones are used in Nature for covalent catalysis such as the quinone cofactors tryptophan tryptophylquinone (TTQ)^42−44^ and topaquinone (TPQ)^45^ (Figure 1C). These cofactors are attacked by substrates to initiate redox catalysis, and the quinone generated by BhaC_1_ can likewise function as an electrophilic handle. Other examples of site-specific enzymatic introduction of ketone/quinone sites are the formation of formylglycine from Cys by the formylglycine-generating enzyme (FGE)^28^ and the recent use of tyrosinase to oxidize Tyr to an *o-*quinone (Figure 1D).^30,31^

**Figure 1:**
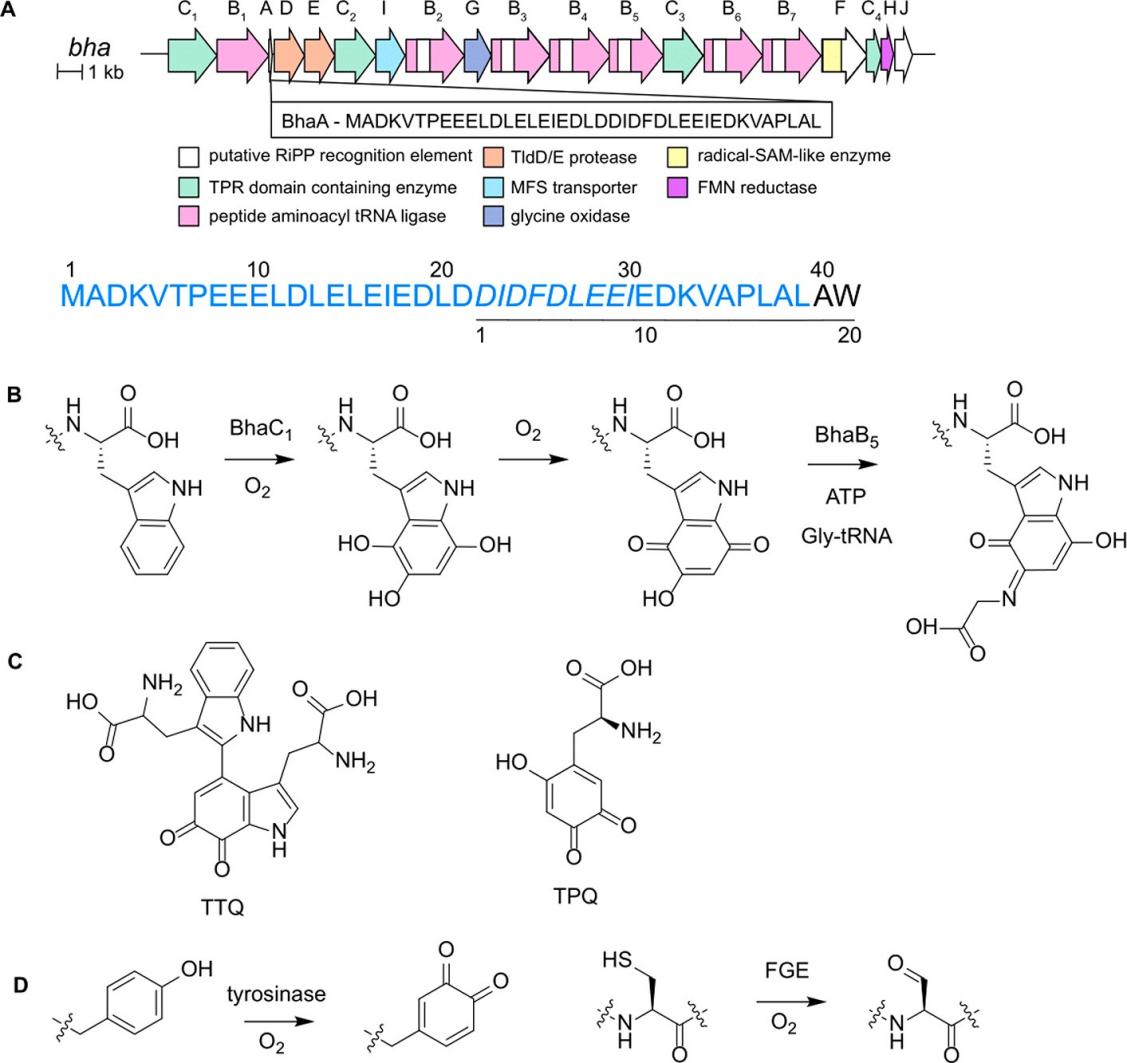
(A) Pearlin biosynthetic gene cluster in *Alkalihalobacillus halodurans* C-125, which encodes the enzyme BhaC_1_ involved in the production of a pyrroloiminoquinone-type natural product. Also shown is the full length precursor scaffold peptide BhaA-Ala-Trp. The Ala and Trp (black) are added by PEARLs to the scaffold peptide BhaA (blue). The 20-amino acid peptide that serves as a substrate for BhaC_1_ in this study is underlined. The nine amino acids termed the recognition residues are in italics. (B) Formation of an *o*-hydroxy-*p*-quinone by BhaC_1_ during pyrroloiminoquinone biosynthesis. BhaC_1_ acts on BhaA-Ala-Trp, hydroxylating the indole three times. The trihydroxylated product undergoes further oxidation to generate the 5-hydroxy-*p*-quinone structure, which is the substrate for BhaB_5_. BhaB_5_ adds glycine in a tRNA^Gly^-dependent reaction. Subsequent decarboxylation and hydrolysis of the appended glycine yield an amino-substituted indole, a core scaffold for pyrroloiminoquinone-type natural products. (C) Structures of the quinone cofactors TTQ and TPQ. (D) Tyrosinase-mediated production of *o*-quinone and aldehyde generation by formylglycine-generating enzyme (FGE).

For chemoenzymatic bioconjugation to be selective in the context of the cellular proteome, catalysis by BhaC_1_ would need to be sufficiently selective not to oxidize Trp at unwanted positions or proteins, yet have sufficient substrate tolerance to act on noncognate proteins and peptides. Trp is the least abundant amino acid in the cell, and one of the rarest in the proteome.^46^ While Trp is scarce, there are still 1195 human protein sequences that contain a C-terminal Trp residue (1.5% of the predicted human proteome). Thus, an enzyme that oxidizes a C-terminal Trp in a sequence-specific manner and that will not modify internal Trp residues would be required for use as a catalyst for site-selective introduction of a modification handle. In this work, we investigated the substrate selectivity of BhaC_1_ with respect to its peptide substrate. Our studies reveal BhaC_1_ has a high level of substrate discrimination and recognizes a partly acidic, partly hydrophobic peptide sequence of 20 amino acids (Figure 1A). That sequence can be appended onto larger proteins to direct *o*-hydroxy-*p*-quinone formation to the fusion protein.

At the time of the first report on BhaC_1_ activity, little bioinformatic information and no structural information were available. Protein Basic Local Alignment Search Tool (BlastP) queries^47−49^ returned proteins of unknown function and identified a TPR domain. Our previous structural predictions on BhaC_1_ utilizing Phyre2 were consistent with the presence of a TPR domain,^38^ but several stretches of the protein remained unstructured. In this work, we used AlphaFold^50^ and AlphaFold Multimer^51^ analysis of BhaC_1_ and its substrate, BhaA-Ala-Trp (Figure 1A). The AlphaFold analysis predicted several structural regions that the Phyre2 model did not contain. Notably, in the AlphaFold model, a positively charged cleft and tunnel is evident with a potential FMN binding site at one end of the tunnel. AlphaFold Multimer predicts BhaA-Ala-Trp interacts with the positively charged cleft and inserts into the tunnel.

## Materials and Methods

### Plasmid Construction and Expression of BhaA Mutants and MBP-Tagged BhaA Constructs

The BhaA mutant expression vectors were constructed using gBlocks (Table S1). The vector backbone and genes were amplified using the polymerase chain reaction (PCR), where the primers contained overlapping regions for Gibson assembly^52^ ligation to generate the plasmid constructs. Plasmids encoding maltose binding protein (MBP) conjugates with a cleavable tobacco etch virus (TEV) protease cleavage site, pET28b-His_6_-MBP-His_6_-TEV-BhaA-10mer, pET28b-His_6_-MBP-His_6_-TEV-BhaA-20mer, and subsequent mutants were constructed using RxnReady primers from Twist with overlapping regions to pET28b-His_6_-MBP-His_6_-TEV (Table S2). The vector backbone was amplified using PCR, where primers contained overlapping regions to the RxnReady primers. Gibson assembly was used to prepare the plasmid constructs. Chemically competent *Escherichia coli* DH5α cells were transformed with the constructed plasmids, and plasmids isolated from the resulting transformants were sequenced and then utilized for expression. All peptides and proteins were expressed in *E. coli* BL21 (DE3) cells. A single colony was used to inoculate 5 mL of Luria-Bertani medium (LB) that was grown with 50 μg/mL kanamycin, or 25 μg/mL kanamycin and 50 μg/mL carbenicillin for co-expression with pET15b-BhaC_1_, overnight at 37 °C with shaking (220 rpm). Then 50 mL of LB was inoculated with 500 μL of the overnight culture and grown with 50 μg/mL kanamycin, or 25 μg/mL kanamycin and 50 μg/mL carbenicillin for co-expression with pET15b-BhaC_1_. At OD_600_ values of 0.6–0.9, cells were induced with 0.3 mM isopropyl β-d-1-thiogalactopyranoside (IPTG) and incubated at 18 °C for 18 h. Cells were harvested by centrifugation at 4500*g* for 10 min at 4 °C.

### Purification of BhaA Mutants and MBP-Tagged BhaA Constructs

Cell pellets were resuspended in 1 mL of lysis buffer [50 mM HEPES and 100 mM NaCl (pH 7.5)] and lysed by the addition of 1 mg/mL lysozyme and sonication (1 s pulse, 1 s pause, 45 s pulse time, 50% amplitude). Insoluble cellular matter was removed by centrifugation at 50000*g* for 1 h at 4 °C, and the supernatant was incubated with Ni-NTA resin for 1 h at room temperature. The lysate/resin mixture was applied to a Bio-Rad Micro Bio-Spin column, and the lysate was pushed through the column by centrifugation at 800*g* for 1 min. The resin was washed with 10 column volumes (CVs) of lysis buffer and 10 CVs of wash buffer [50 mM HEPES, 100 mM NaCl, and 25 mM imidazole (pH 7.5)]. The peptides or MBP-tagged peptides were eluted with 500 μL of elution buffer [50 mM HEPES, 100 mM NaCl, and 500 mM imidazole (pH 7.5)]. The production of MBP-tagged peptides was validated using sodium dodecyl sulfate–polyacrylamide gel electrophoresis (Figure S1).

### Mass Spectrometric Analysis of BhaA Mutants

All purified peptides were analyzed using matrix-assisted laser desorption ionization time-of-flight mass spectrometry (MALDI-TOF MS). Samples were desalted by ZipTip using Agilent C18 tips, eluted with 80% acetonitrile and 0.1% trifluoroacetic acid (TFA), and spotted onto a MALDI plate with Super 2,5-dihydroxybenzoic acid (SuperDHB) matrix [9:1 (w/w) mixture of 2,5-DHB and 2-hydroxy-5-methoxybenzoic acid]. MBP-tagged peptides were first subjected to TEV cleavage to free the C-terminal peptide from MBP before mass spectrometry sample preparation and assessment.

### Structure Prediction Using AlphaFold

The wild-type (WT) sequences for BhaC_1_ and AmmC_1_ were obtained from NCBI and used for structural prediction with AlphaFold 2.1.2.^50^ WT BhaC_1_ (listed first in the fasta submission file) and His_6_-BhaA-Ala-Trp (listed second) (GSSHHHHHHSQDPMADKVTPEEELDLELEIEDLDDIDFDLEEIEDKVAPLALAW) sequences were submitted for structural prediction of the 1:1 heterodimer in AlphaFold Multimer version 2.1.2. WT AmmC_1_ (listed second), and His6-AmmA*-Trp (GSSHHHHHHSQDPMSETQVTETDNPAEEPAEIAAESDDLADLDDIEFDLDEVESKIAPLALAW) (listed first) sequences were submitted for structural prediction of the 1:1 heterodimer in AlphaFold Multimer^51^ version 2.1.2. Structures were visualized using ChimeraX.^53^

## Results

### BhaC_1_ Modifies only a C-Terminal Trp

BhaC_1_-catalyzed modification of BhaA-Ala-Trp was previously investigated *in vitro* and by co-expression in *E. coli*.^38^ The enzyme activity is generally better in *E. coli*, possibly because of the presence of flavin reductases. In the previous study, co-expression of BhaC_1_ with its substrate BhaA-Ala-Trp resulted in a 48 Da increase of the C-terminal Trp residue as confirmed by tandem mass spectrometry (Figure 1A,B).^38^ Nuclear magnetic resonance characterization demonstrated the formation of an *o*-hydroxy-*p*-quinone on the indole (Figure 1B). To explore whether BhaC_1_ would modify a Trp that was not the C-terminal residue, we generated the mutants BhaA-Ala-Trp-Ala and BhaA-Ala-Trp-Gly. Upon co-expression with BhaC_1_, we observed little to no modification (Figure S2).

### The Identity of the Amino Acids N-Terminal to the Trp Is Important

To investigate the substrate specificity toward residues N-terminal to the Trp, we designed constructs to generate His_6_-BhaA-Xxx-Trp variants to evaluate if BhaC_1_ would tolerate substitutions of the native penultimate Ala. In a multiple-sequence alignment of BhaA homologues (identified by BlastP),^47,48^ this Ala directly N-terminal to Trp is conserved among many of the sequences (Figure S3). This Ala residue is missing in BhaA but is installed via PEARL-catalyzed addition of the amino acid by BhaB_1_.^38^ We replaced Ala with representative amino acids from each structural group: charged (Asp and Lys), uncharged polar (Asn, Ser, and Thr), conformationally restricted (Pro and Gly), and hydrophobic [Val, Phe, and Trp (for sequences, see Figure S4)]. For the charged amino acids, neither His_6_-BhaA-Asp-Trp nor His_6_-BhaA-Lys-Trp was accepted by BhaC_1_ (Figure S4), and for the uncharged polar amino acids, neither His_6_-BhaA-Thr-Trp nor His_6_-BhaA-Asn-Trp was modified. However, partial modification was observed for His_6_-BhaA-Ser-Trp (+16, +32, and +48 Da products) (Figure S4). For variants substituted with amino acids that have either conformational restriction (Pro) or flexibility (Gly), modification was observed only for His_6_-BhaA-Gly-Trp (+16, +32, and +48 Da products observed) (Figure S4). For the hydrophobic amino acid variants His_6_-BhaA-Val-Trp, His_6_-BhaA-Phe-Trp, and His_6_-BhaA-Trp-Trp, no modification was observed (Figure S4). Thus, BhaC_1_ is quite selective with respect to the residue flanking the C-terminal Trp.

### BhaC_1_ Can Modify Tagged Proteins

**Figure 2:**
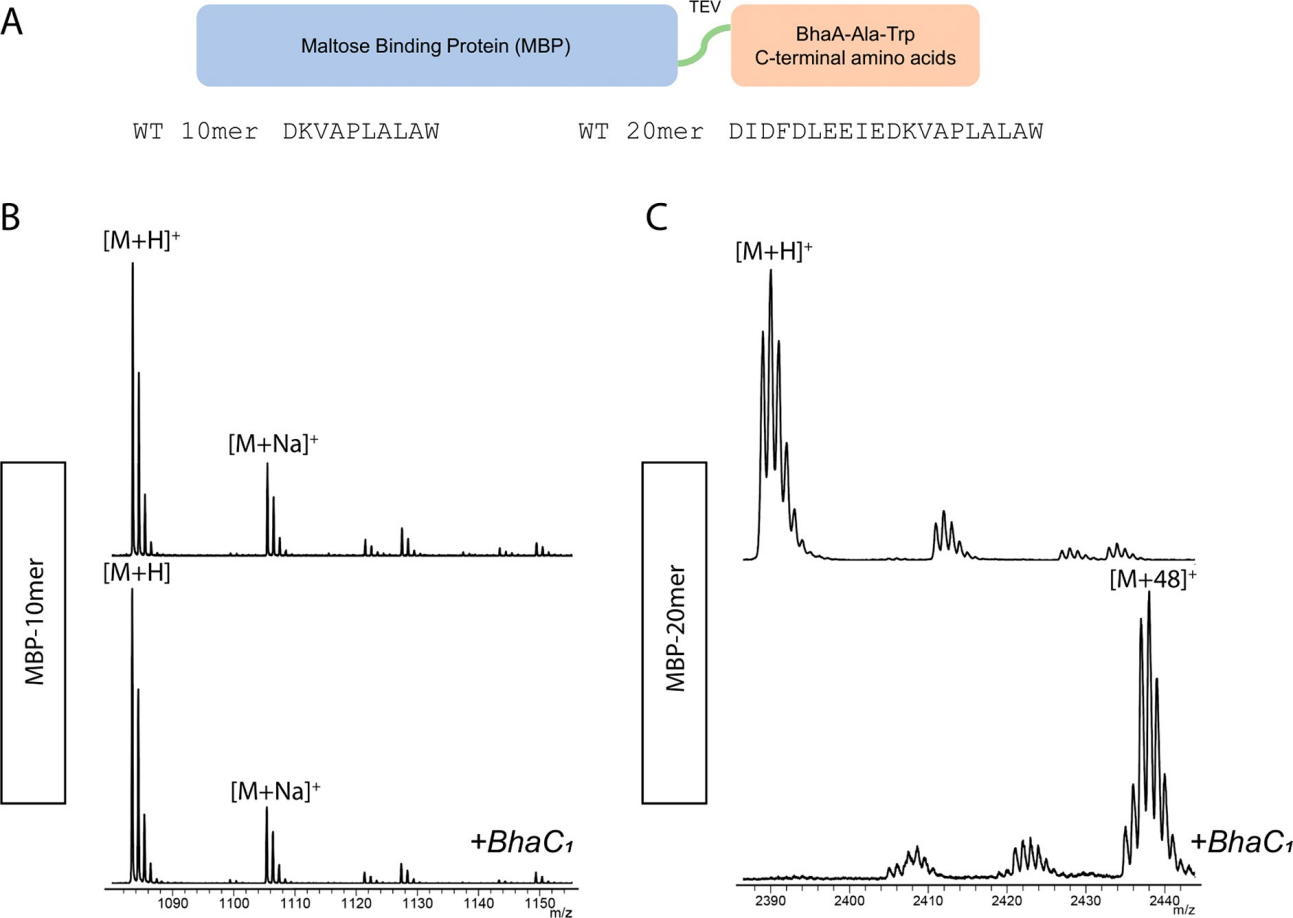
MALDI-TOF MS of TEV-cleaved His_6_-MBP-His_6_-TEV-BhaA-10mer and -20mer proteins co-expressed with BhaC_1_ in *E. coli*. The 10mer peptide was not accepted by BhaC_1_, while the 20mer sequence was almost fully modified to the +48 Da product. (A) Schematic of the MBP–peptide conjugates investigated. (B) MALDI-TOF MS of MBP-10mer expressed in *E. coli* after TEV cleavage (top) (calculated *m*/*z* 1083.6, observed *m*/*z* 1083.5) and MALDI-TOF MS of MBP-10mer co-expressed with BhaC_1_ in *E. coli* after TEV cleavage (bottom). (C) MALDI-TOF MS of MBP-20mer expressed in *E. coli* after TEV cleavage (top) (calculated *m*/*z* 2389.2, observed *m*/*z* 2389.0) and MALDI-TOF MS of MBP-20mer co-expressed with BhaC_1_ in *E. coli* after TEV cleavage (bottom) (calculated [M + 3O + H]^+^*m*/*z* 2437.1, observed *m*/*z* 2436.9). ). Some product peptides show additional ions at M − 2 Da. These are the *o*-hydroxyquinone structures that form spontaneously (Figure 1B).

After establishing that BhaC_1_ modifies C-terminal Trp and does not tolerate most substitutions to the amino acid N-terminal to the Trp, we explored the minimal substrate for catalysis. We elected to investigate this question in the context of a fusion protein rather than synthetic peptides because the desired application of BhaC_1_ would involve appending the minimal sequence to proteins of interest. Therefore, we generated constructs in which C-terminal sequences of BhaA-Ala-Trp of various lengths were fused to the C-terminus of maltose binding protein (MBP) with a TEV cleavage site between MBP and the C-terminal sequence (Figure 2A). We started with the C-terminal 10mer (DKVAPLALAW) and 20mer (DIDFDLEEIEDKVAPLALAW) sequences of BhaA-Ala-Trp that were encoded in plasmids that contained His-tagged MBP reported previously.^54^ The resulting His_6_-MBP-His_6_-TEV-10mer and -20mer proteins were co-expressed with BhaC_1_ in *E. coli* (Figure 2A−C). The MBP-10mer and -20mer proteins were purified and treated with TEV protease. The C-terminal 10mer and 20mer peptides were then analyzed by MALDI-TOF MS. His_6_-MBP-His_6_-TEV-20mer was almost completely modified to the +48 Da product (Figure 2C). In contrast, His_6_-MBP-His_6_-TEV-10mer was not a substrate (Figure 2B). Therefore, the minimal substrate is between 10 and 20 amino acids in length.

### The First Nine Amino Acids of the C-Terminal 20mer Are Critical for Modification

**Figure 3:**
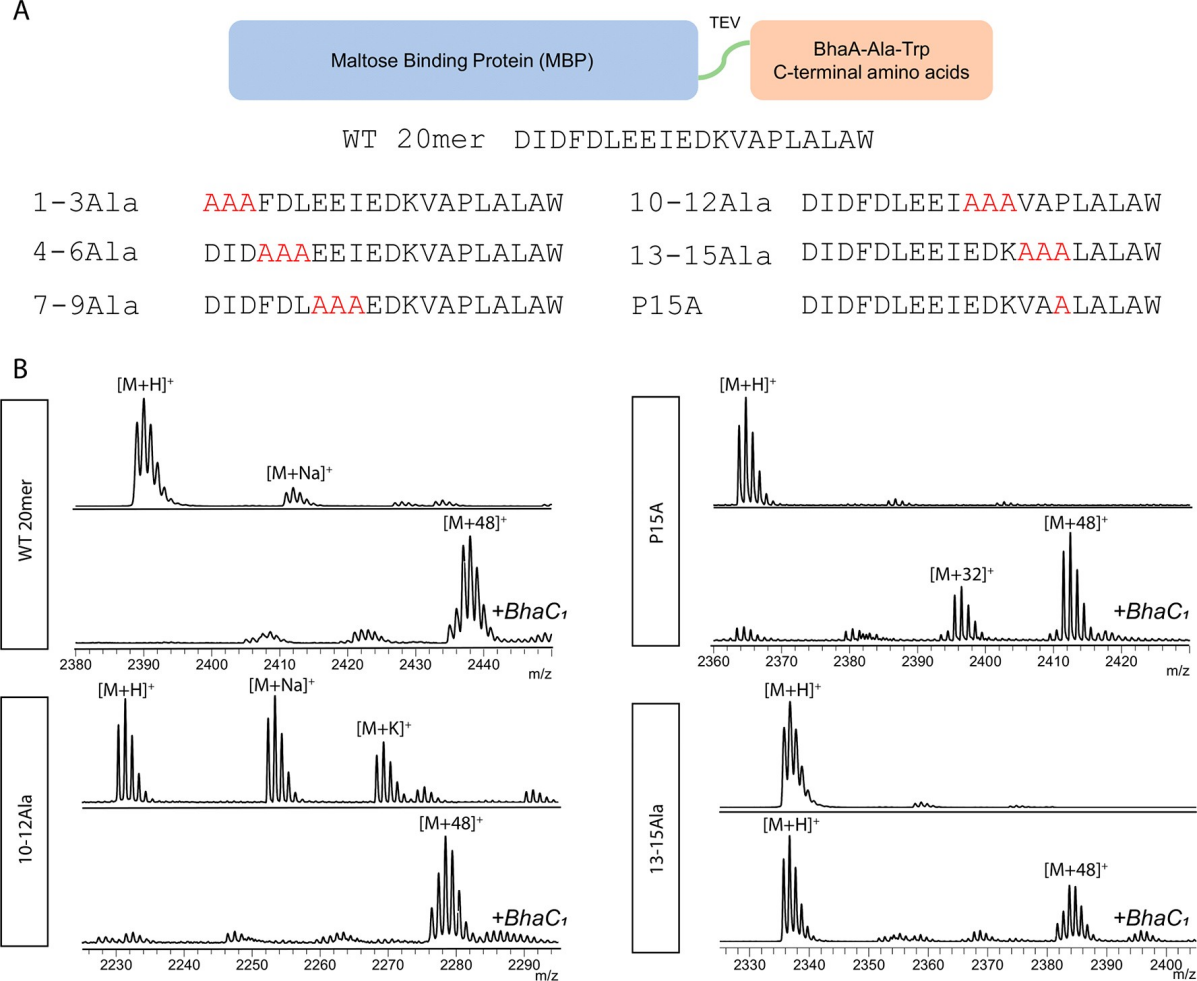
Alanine scanning of MBP-20mer substrates. Variants were generated to test substrate recognition and modification by BhaC_1_. (A) Schematic of MBP peptide conjugates. Mutated residues are colored red. (B) MALDI-TOF MS of variants of the MBP-20mer substrate after expression without (top) and with (bottom) BhaC_1_. Data are shown for variants that were still accepted to varying extent by BhaC_1_. All MBP–peptide conjugates were subjected to TEV cleavage to free the 20mer peptide before MALDI-TOF MS analysis. Some product peptides show additional ions at M^–^ 2 Da. These are the *o*-hydroxyquinone structures that form spontaneously (Figure 1B). For calculated and observed masses, see Table S3.

To investigate the importance of the residues in the C-terminal 20mer for BhaC_1_ catalysis, a series of alanine mutants were generated where sets of three residues were simultaneously substituted with Ala in the context of the MBP fusion protein (Figure 3A). In all variants of the 20mer in this study, we number the residues 1–20, which is different from the numbering in full length BhaA (for conversion to BhaA numbering, see Figure 1A). His_6_-MBP-His_6_-TEV-20mer-D1A/I2A/D3A (designated 1–3Ala), His_6_-MBP-His_6_-TEV-20mer-F4A/D5A/L6A (4–6Ala), and His_6_-MBP-His_6_-TEV-20mer-E7A/E8A/I9A (7–9Ala) were not modified (Figure S5). We termed these nine amino acids the recognition residues. Conversely, co-expression of His_6_-MBP-His_6_-TEV-20mer-E10A/D11A/K12A (10–12Ala) with BhaC_1_ led to nearly complete modification, and co-expression of His_6_-MBP-His_6_-20mer-V13A/A14A/P15A (13–15Ala) with BhaC_1_ led to partial modification (Figure 3B). Therefore, the first nine amino acids and the last two residues in the 20mer sequence (Ala-Trp) appear to be critical for modification. The 20mer sequence was used in all subsequent MBP fusion studies that investigated substrate specificity in more detail.

### Pro15 Is Not Important, but Leu18 of BhaA-Ala-Trp C-Terminal 20mer Is Essential for Modification

To investigate the importance of Pro15 in the C-terminal 20mer sequence, another highly conserved residue (Figure S3) that could be important for the conformation of the peptide, we prepared His_6_-MBP-His_6_-TEV-20mer-P15A (Figure 3A). This variant was almost completely modified by BhaC_1_ (Figure 3B), illustrating that Pro15 is not critical despite its conservation in homologues. We also investigated the importance of Leu18 of the C-terminal 20mer, located two residues from the C-terminal Trp in BhaA-Ala-Trp. This residue is highly conserved among homologues (Figure S3). We generated four variants, His_6_-MBP-His_6_-TEV-20mer-L18A, -L18E, -L18F, and -L18K. None of these were substrates for BhaC_1_ (Figure S6). Thus, the enzyme has high specificity for the two residues (Leu-Ala) preceding the C-terminal Trp.

### The Distance between the Recognition Residues and Trp Is Important

**Figure 4:**
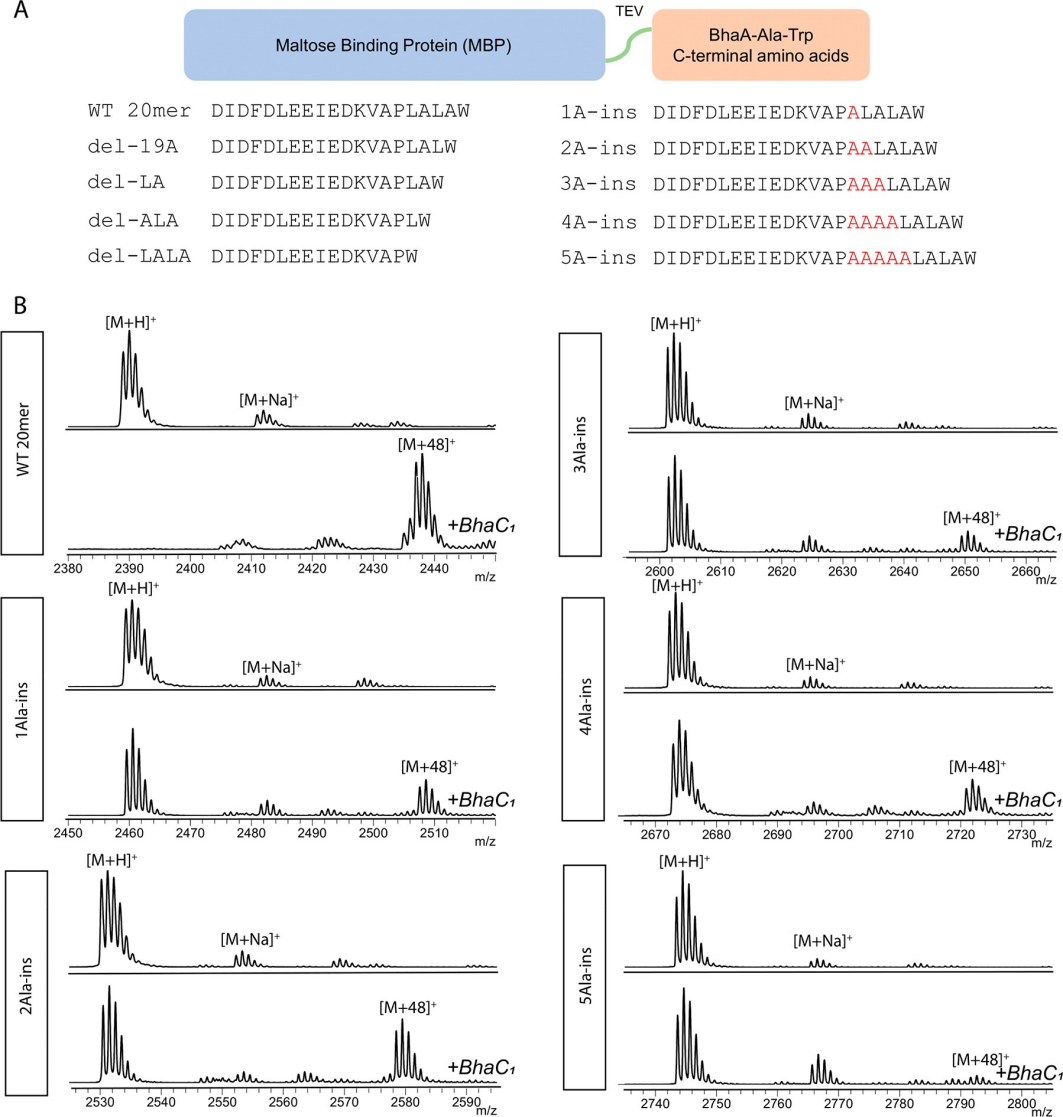
Deletions and insertions in the MBP-20mer. Variants were generated to test substrate recognition and modification by BhaC_1_. (A) Schematic of MBP–peptide conjugates. Deletions moved the C-terminal Trp closer to and insertions farther from the nine-amino acid recognition sequence in the 20mer sequence. Insertions are colored red. (B) MALDI-TOF MS of deletion and insertion mutants of the MBP-20mer substrate expressed with and without BhaC_1_. Spectra are shown for variants that were still accepted to varying extent by BhaC_1_. MBP conjugates were subjected to TEV cleavage and then analyzed via MALDI-TOF MS. For calculated and observed masses, see Table S3.

To examine the importance of the distance between the recognition residues identified above that are critical for modification and the Trp residue to be modified, we generated variant sequences by insertion of Ala residues between Pro15 and Leu16 of the 20mer to increase the distance between the Trp and the recognition residues (Figure 4A). For His_6_-MBP-His_6_-TEV-20mer-*ins*1A, His_6_-MBP-His_6_-TEV-20mer-*ins*2A, His_6_-MBP-His_6_-TEV-20mer-*ins*3A, and His_6_-MBP-His_6_-TEV-20mer-*ins*4A, low to moderate conversion to the +48 Da product was observed (Figure 4B). For His_6_-MBP-His_6_-TEV-20mer-*ins*5A, minimal conversion to the trihydroxylated product was detected (Figure 4B). We also designed mutants in which residues in the LALA motif were successively deleted to bring the Trp residue closer to the recognition residues (Figure 4A). His_6_-MBP-His_6_-TEV-20mer-ΔA19 His_6_-MBP-His_6_-TEV-20mer-ΔL18/ΔA19, His_6_-MBP-His_6_-TEV-20mer-ΔA17/ΔL18/ΔA19, and His_6_-MBP-His_6_-TEV-20mer-ΔL16/ΔA17/ΔL18/ΔA19 were not accepted as substrates (Figure S7). These findings support the hypothesis that the 20mer is the minimal substrate and that the distance between the N-terminal recognition residues in this sequence and the C-terminal Trp is important for modification. Extending this distance is moderately tolerated, but shortening the distance abolished activity. Hence, it appears that the intervening stretch of amino acids between the recognition sequence and the C-terminal Trp is required for the latter to reach the active site.

### Structural Prediction of BhaC_1_ and Its Complex with Bha-Ala-Trp

**Figure 5:**
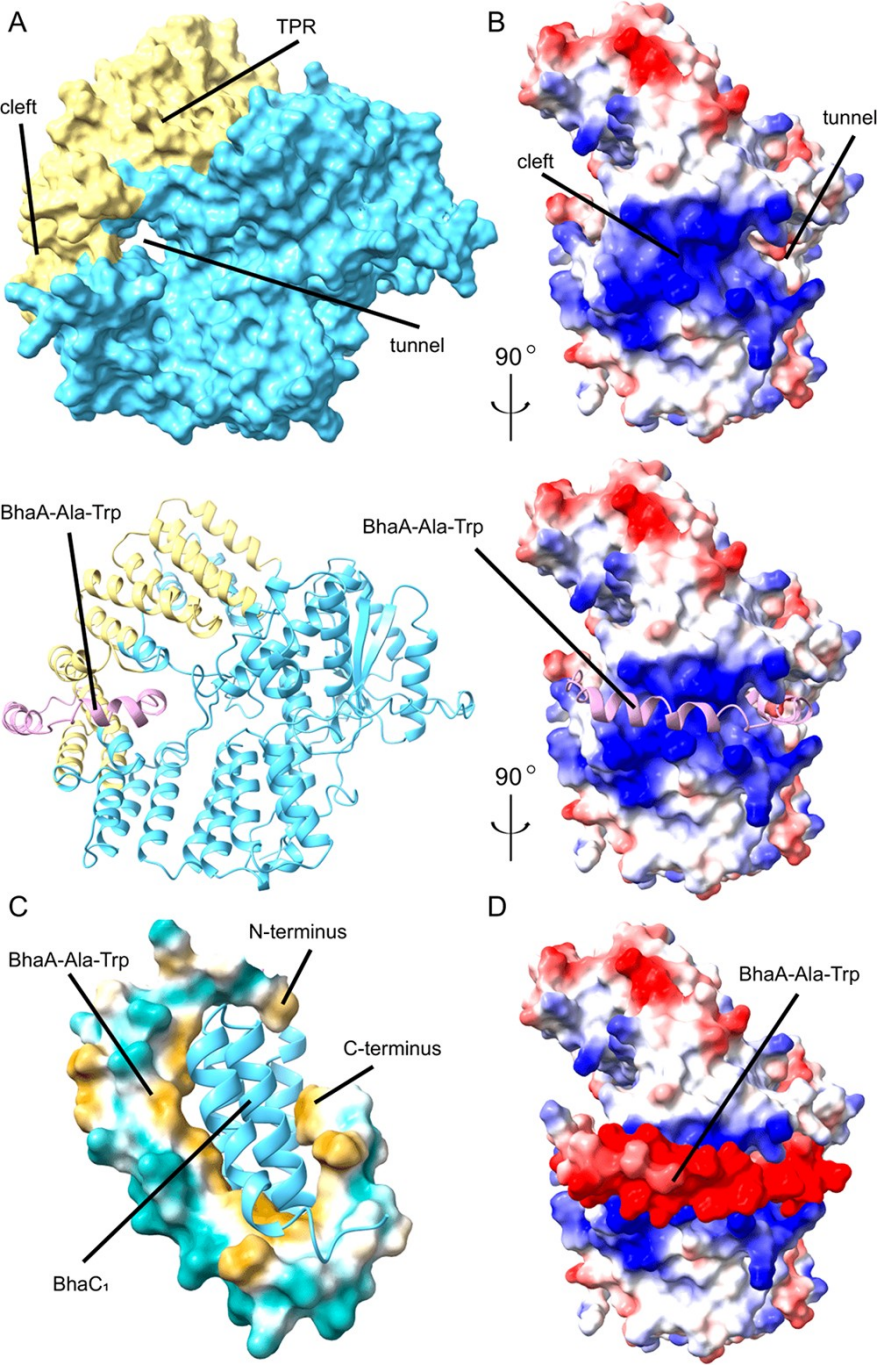
AlphaFold Multimer analysis of BhaC_1_ and its substrate BhaA-Ala-Trp. The AlphaFold analysis was conducted with His_6_-BhaA-Ala-Trp as the substrate. (A) BhaC_1_ is colored blue, the TPR domain yellow, and BhaA-Ala-Trp pink. The AlphaFold prediction features a tunnel; the Multimer algorithm predicts that the C-terminus of BhaA-Ala-Trp binds in this tunnel. (B) BhaC_1_ space-filling rendition illustrating electrostatics. The positively charged cleft in the TPR domain binds the helical and negatively charged N-terminal portion of BhaA-Ala-Trp. (C) Hydrophobicity plot of the interaction of Bha-Ala-Trp with BhaC_1_ according to the Kyte–Doolittle scale. Orange-yellow indicates hydrophobic, teal hydrophilic, and white neutral. AmmC_1_ is colored blue. The side of the Bha-Ala-Trp α-helix and the subsequent turn that faces BhaC_1_ entirely consist of hydrophobic amino acids. (D) The negatively charged amino acids on BhaA-Ala-Trp face outward but make extensive interactions with the positively charged side chains of Lys and Arg residues on BhaC_1_ (see Figure 6). For confidence values for the individual amino acids in the model, see Table S4. Figure made using ChimeraX.^53^

**Figure 6:**
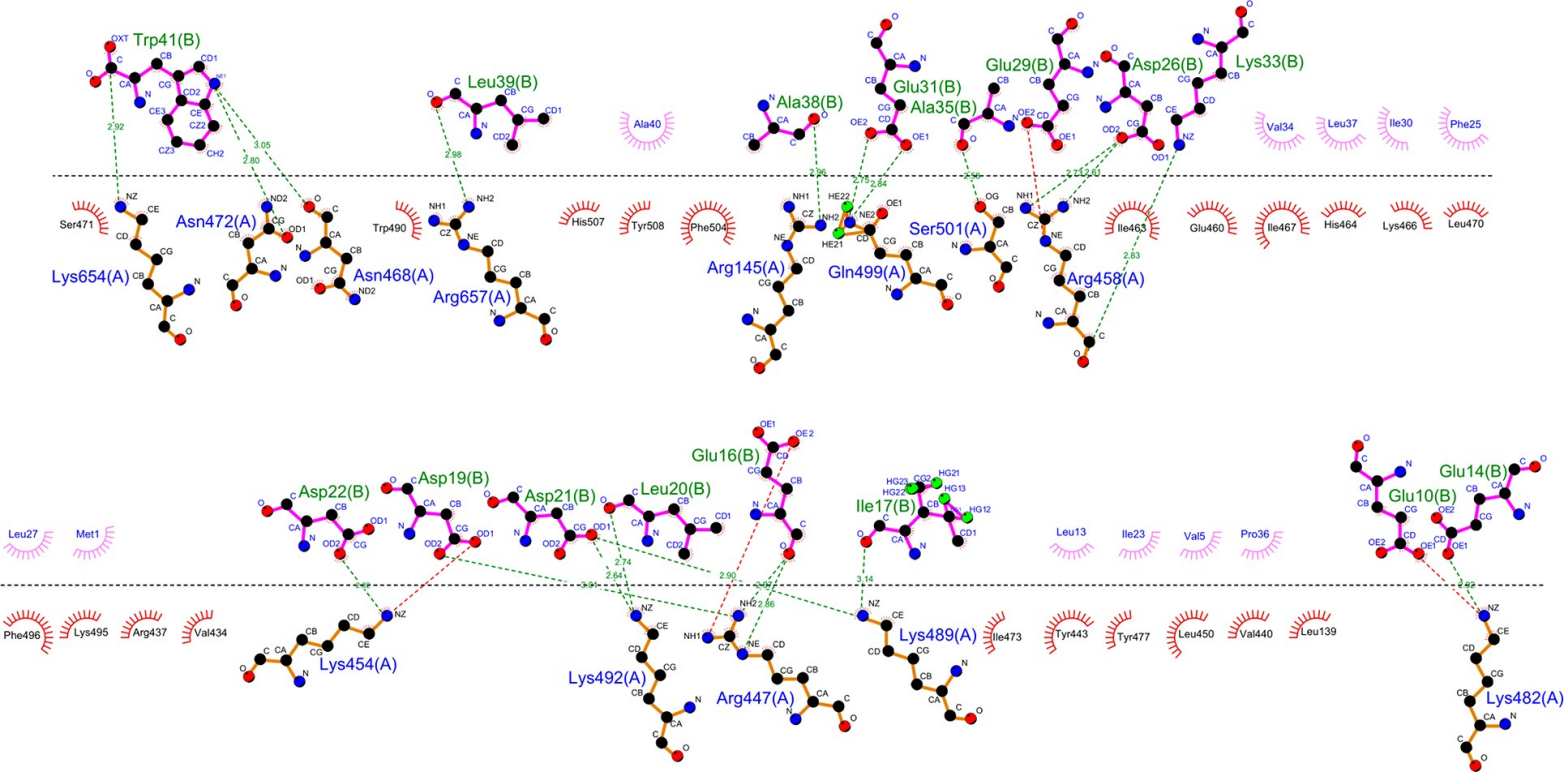
DimPlot illustrating interactions between BhaC_1_ and its substrate BhaA-Ala-Trp. BhaC_1_ is sequence (A), and BhaA-Ala-Trp is sequence (B). BhaA-Ala-Trp was submitted as the full length sequence for DimPlot analysis (Met1–Trp41). For the residues that make hydrophobic contacts shown in the half-circles, residues above the dotted line are from sequence B (BhaA-Ala-Trp) and residues below the dotted line are from sequence A (BhaC_1_).

We first utilized AlphaFold 2.0^50^ to predict a model for apo-BhaC_1_. No obvious flavin binding domain was observed even though BhaC_1_ is purified with bound FMN.^38^ In the model, a tunnel and a potential FMN coordination site are next to the predicted TPR domain (Figure 5A), which has a highly positively charged, surface-exposed cleft (Figure 5B). The scaffold peptides for the biosynthesis of known PEARL-mediated pyrroloiminoquinone natural products are highly negatively charged (Figure S3).^38^ Multiple-sequence alignment of BhaC_1_ with homologues from diverse phyla (identified using BlastP)^48,49^ revealed that many of the amino acids in and near the tunnel and the positively charged cleft are highly conserved (Figure S8). To provide a visual approximation of the BhaC_1_–substrate complex, AlphaFold Multimer^51^ was used to predict a structure of the 1:1 heterodimer of BhaC_1_ and His_6_-Bha-Ala-Trp (Figure 5). The predicted model suggests that the negatively charged N-terminus of BhaA-Ala-Trp engages the positively charged cleft within the TPR domain of BhaC_1_ as an α helix (Figure 5 and Figure S9). This helix starts at Pro7 and ends at Leu20 (BhaA numbering), followed by a turn from Asp21 to Asp32 after which the C-terminal part of BhaA inserts into the tunnel (Figure 5A–C). The face of the helix that is oriented toward BhaC_1_ is hydrophobic and contains Leu13, Ile17, and Leu20 (Figure 5C and Figure S9B). Similarly, the turn sequence contains hydrophobic amino acids (Ile23, Phe25, Leu27, and Ile30) that are making van der Waals contacts with the enzyme (Figure S9B). The negatively charged amino acids on BhaA-Ala-Trp are mostly on the side of the helix (Glu8, Glu9, Glu10, Glu14, Glu16, Glu18, and Asp19) and turn structure (Asp21, Asp22, Asp24, Asp26, Glu28, Glu31, and Asp32) that faces away from BhaC_1_ (Figure 5C,D), but the side chains make numerous interactions with the positively charged side chains of residues on the enzyme, as illustrated schematically in the DimPlot^55^ rendition in Figure 6. The DimPlot software (similar to LigPlot)^55^ generates schematic diagrams of interactions across protein–protein or domain–domain interfaces for a given Protein Data Bank file. Here, the AlphaFold Multimer output was submitted to the DimPlot software.

AmmC_1_, a BhaC_1_ homologue utilized in ammosamide biosynthesis in *Streptomyces* sp. CNR-698, also contains a TPR domain^38^ and also trihydroxylates the indole of the C-terminal Trp in its substrate AmmA*-Trp.^56^ AlphaFold predicts that the AmmC_1_ TPR domain also has a positively charged cleft that binds AmmA*-Trp and a tunnel that houses the C-terminus of the substrate with the C-terminal Trp (Figure S10). The α-helix of AmmA*-Trp is not as well-defined, and the confidence levels for the individual amino acids are lower than for the BhaA-Ala-Trp-BhaC_1_ complex; however, many of the interactions of BhaA-Ala-Trp with BhaC_1_ are conserved in the predicted interaction of AmmC_1_ with AmmA*-Trp (Figures S10–S13). The indole nitrogen of the C-terminal Trp of the substrates interacts with a conserved Asn in both models (Asn472 in BhaC_1_ and Asn432 in AmmC_1_), and a pair of Lys residues (Lys391/654 in BhaC_1_ and Lys350/602 in AmmC_1_) interact with the C-terminal carboxylate (Figure 6 and Figure S13). Because FMN was not present in the AlphaFold model, the details of the interactions of the C-terminal Trp with the enzymes require a crystal structure of a complex, which is not available at present. Regardless, the side chain of the Trp cannot be bound too rigidly because the enzyme performs three hydroxylations that require some movement of the indole.

Many of the direct interactions between BhaC_1_ and BhaA-Ala-Trp are in the C-terminal 20mer sequence, which includes the turn sequence as well as the C-terminus that is inserted into the tunnel. The predicted binding mechanism agrees well with the results of the biochemical studies providing an increased level of confidence in the model. The nine residues identified as the recognition residues in the minimal substrate cover almost the entire turn sequence just before the C-terminal sequence inserts into the tunnel. The similar interactions between the orthologous pairs of peptides and enzymes in the bha and amm pathways suggest a common extended binding motif that uses the cleft in the TPR domain for recognition and affinity and guides the catalytically important C-terminus through the tunnel into the active site. This model also explains the importance of the length of the sequence between the recognition residues and the C-terminal Trp as a shorter peptide would not be able to reach the active site and longer peptides are much poorer substrates.

## Summary

The findings from the current study reveal that BhaC_1_ is a highly specific enzyme capable of performing three hydroxylations on the indole of a C-terminal Trp residue that is at a defined distance from a stretch of nine recognition residues in a 20-amino acid peptide. A theoretical model provides a potential explanation for the observed sequence recognition. Introduction of this peptide at the C-terminus of maltose binding protein resulted in initial trihydroxylation of the indole of the C-terminal Trp followed by spontaneous oxidation to the corresponding *o*-hydroxy-*p*-quinone,^38^ yielding an electrophilic handle for bioconjugation. In the realm of organic chemistry, many examples have been reported of such quinones reacting with amines,^57^ phosphines,^58^ and thiols.^57,58^ On the basis of the well-documented use of other structurally related quinones for bioconjugation and its high substrate specificity, BhaC_1_ is a promising candidate for attachment of designer molecules to proteins of interest. Utilizing the BhaC_1_ product for bioconjugation of target proteins will be a topic for future investigation.

## Supporting Information Available

Figures S1–S13 and Tables S1–S4 with primer and gene sequences, masses of observed ions, and confidence values for AlphaFold models (PDF)

## Accession Codes

NCBI accession numbers: BhaC_1_, BAB05752.1; AmmC_1_, WP_051437089.1; BhaA, BAB05754.1.

## Abbreviations

CV: column volume
FMN: flavin mononucleotide
HEPES: 4-(2-hydroxyethyl)piperazine-1-ethanesulfonic acid
HPLC: high-performance liquid chromatography
IMAC: immobilized metal affinity chromatography
IPTG: isopropyl β-d-1-thiogalactopyranoside
LB: Luria-Bertani medium
MALDI: matrix-assisted laser desorption ionization
MBP: maltose binding protein
MS: mass spectrometry
NTA: nitrilotriacetic acid
PEARL: peptide aminoacyl-tRNA ligase
RiPP: ribosomally translated and posttranslationally modified peptide
TEV: tobacco etch virus
TFA: trifluoroacetic acid
TOF: time-of-flight
DHB: 2,5-dihydroxybenzoic acid.
