# Supplementary material for: Substrate Specificity of the Flavoenzyme BhaC1 That Converts a C-Terminal Trp to a Hydroxyquinone
Source: Biochemistry. 2022 May 25;62(2):378–87. doi: 10.1021/acs.biochem.2c00206 (PMC9850906; doi:10.1021/acs.biochem.2c00206)
Supplement: Supplementary file 1 — bi2c00206_si_001.pdf [file bi2c00206_si_001.pdf]

Supplementary Information

Substrate specificity of the flavoenzyme BhaC$_1$ that converts a C-terminal Trp to a hydroxyquinone

Page N. Daniels[1] and Wilfred A. van der Donk[1-3*]

[1] Department of Biochemistry, University of Illinois at Urbana-Champaign, Urbana, Illinois 61801, USA. [2] Department of Chemistry and Howard Hughes Medical Institute, University of Illinois at Urbana-Champaign, Urbana, Illinois 61801, USA. [3] Carl R. Woese Institute for Genomic Biology, University of Illinois at Urbana-Champaign, Urbana, Illinois 61801, USA.

* To whom correspondence should be addressed:

Wilfred A. van der Donk

600 S. Mathews Avenue

Urbana, Illinois 61801, United States

vddonk@illinois.edu

phone: (217) 244-5360

fax: (217) 244-8533

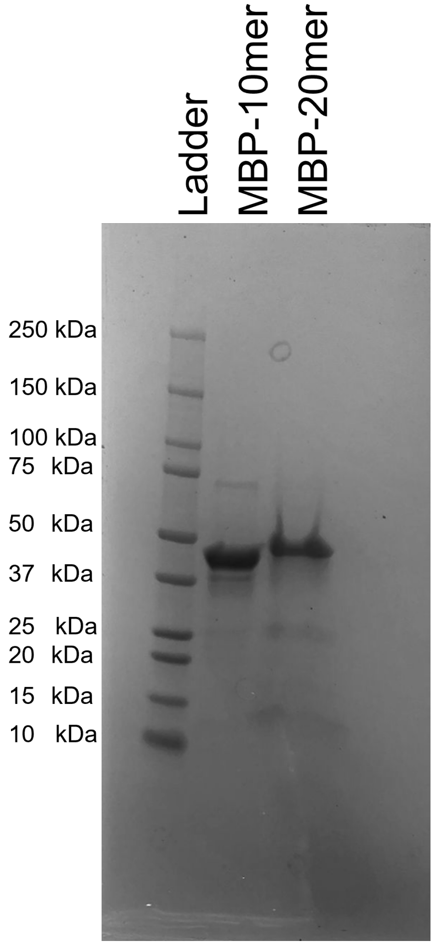

Fig. S1. SDS-PAGE gel of MBP-tagged 10mer and 20mer peptides to verify expression in *E. coli*. MBP-conjugates were purified via Ni-NTA chromatography.

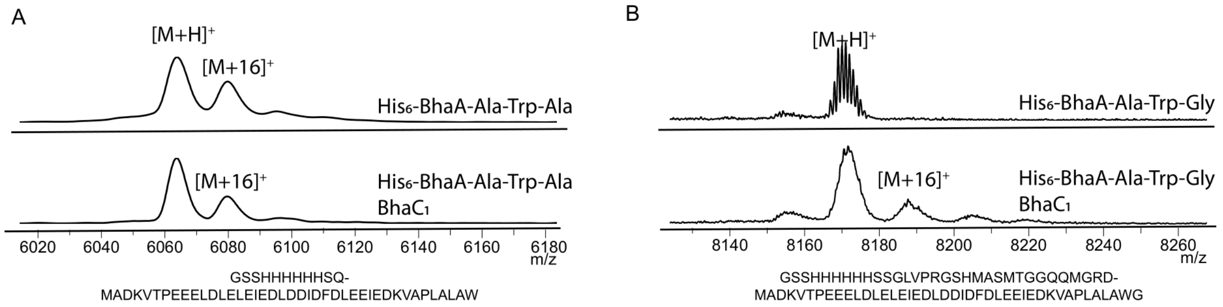

Fig. S2. $BhaC_1$ co-expression with His_6-BhaA-Ala-Trp-Ala and His_6-BhaA-Ala-Trp-Gly. (A) MALDI-TOF MS of His_6-BhaA-Ala-Trp-Ala expressed with and without $BhaC_1$. His_6-BhaA-Ala-Trp-Ala calculated m/z = 6067.8 Da; observed = 6063.2 Da. (B) MALDI-TOF MS of His_6-BhaA-Ala-Trp-Gly expressed with and without $BhaC_1$. His_6-BhaA-Ala-Trp-Gly calculated m/z = 8171.9 Da; observed = 8171.0 Da. The peptides have differences in mass due to variations in amino acid sequence between the His_6-tag and the BhaA peptide sequence. Sequences are indicated below the MS image.

```
BhaA                 ------------------------------------MADKVTPEEELDLELE-IEDLDDIDFDLEEIE-DKVAPLAL----------

WP_147268962.1       ----------------MADEE-IVAE--------------PAAEENEYDDGEELGDLDDMEFLLEEIE-NRIAPLA-----------
WP_223768017.1       ----------------MAEEKTLEET--------------AAAVEAIPEDGEEIDELDDMEFLLEEIE-NRIAPLA-----------
WP_184616655.1       ----------------MADEE-ITET--------------GAAEETDL-DGDELGDLDDMEFLLEEIE-NRIAPLA-----------
WP_028481745.1       --MSNIENSTSNTTEEVREDVS---------NPTDEVKSVDATVQHEENHDD-LDDLDDLDFDLEDVE-DKIAPLAL----------
WP_143043095.1       ----------------MASEETLSAAPAH-----SEPATSEAAHNEVSTAPEKLDEIEEIDFLLEEIE-SKIAPLALA---------
WP_015347328.1       ----------------MELKASESSPVASSTVSEASSAPMELAASEVVPPPEKLDEIEEIDFLLEEIE-SKIAPLALA---------
AKQ68463.1           ----------------MASEESLSR---VAA---DESSVASTAP-DAAAPAEKLDEIEEIDFLLEEIE-SKIAPLALA---------
AKJ01548.1           ----------------------------------MAAPAQATEPHAVSAAPEKLDELEEINFLLEEIE-SKIAPLALA---------
ABF89688.1           ----------------MAPEETLSAAPAH-----SEPATSEAAHSEVSAAPEKLDEIEEIDFLLEEIE-SKIAPLALA---------
WP_163868907.1       ----------------MASEASESVQSPNTSTVVSEAAA-PSAEPSVAAAPEKLDEIEEIDFLLEEIE-SKIAPLALA---------
WP_176416258.1       ----------------MASEDTFQQVHHPVDASSVSAEP-SAPSAEL-AAPEKLDEIEEIDFLLEEIE-SKIAPLALA---------
WP_140788747.1       ----------------MASEDTFQQVHHPVDASSVSAQP-SEPSAELAAAPEKLDEIEEIDFLLEEIE-SKIAPLALA---------
WP_163783181.1       ----------------MASEDTFQQVHHPVDASSVSAQP-SAPSAEL-AAPEKLDEIEEIDFLLEEIE-SKIAPLALA---------
WP_141643698.1       ----------------MASEETAQSPSELAATAVST----STTAEAVSAAPEKLDEIEEIDFLLEEIE-SKIAPLALA---------
WP_046711779.1       ----------------MASEEAVQSPSETAANAVSTPNP-SEAPEAVSAAPEKLDEIEEIDFLLEEIE-SKIAPLALA---------
WP_163995958.1       ----------------MASDGTTPVQAAPSVSSQPA-AE-PMPVEASAAAPEKLDEIEEIDFLLEEIE-SKIAPLALA---------
WP_203407067.1       ----------------MASEESSVSPIESAPSEQQA----QAAAPSLEAAPEKLDELEEINFLLEEIE-SKIAPLALA---------
WP_140861314.1       ----------------MASEET--LSAAPA-HSEPA-TS-EAAHNEASAAPEKLDEIEEIDFLLEEIE-SKIAPLALA---------
WP_206721133.1       ----------------MASDVLTQSPVEPAASATSSAAPAAEPVQAVAAAPEKLDEIEEIDFLLEEIE-SKIAPLALA---------
WP_011551720.1       ----------------MAPEE-----TLSAAPAHSEPATSEAAHSEVSAAPEKLDEIEEIDFLLEEIE-SKIAPLALA---------
WP_141590155.1       ----------------MAPEE-----TLSAAPAHSEPATSEAAHNEVSAAPEKLDEIEEIDFLLEEIE-SKIAPLALA---------
WP_002639333.1       ----------------MASEESLSRVAADESSV-------ASTAPDAAAPAEKLDEIEEIDFLLEEIE-SKIAPLALA---------
WP_123810841.1       ----------------MASQENPLPSTESQVAAPAQ----ATEPHAVSAAPEKLDELEEINFLLEEIE-SKIAPLALA---------
WP_206719380.1       ----------------MALKASESSPVAPSTVSEASSAPMELAASETVPPPEKLDEIEEIDFLLEEIE-SKIAPLALA---------
WP_013935244.1       ----------------MASEET-----LSAAPAQ-------NASATVDAAPEKLDEIEEIDFLLEEIE-SKIAPLALA---------
WP_205610140.1       ----------------------------SAAPAHSEPATSEAAHNEASAAPEKLDEIEEIDFLLEEIE-SKIAPLALA---------
NVJ07078.1           ----------------MASEDTFQQVPHPVDASSVSAQS--SAPSAELAAPEKLDEIEEIDFLLEEIE-SKIAPLALA---------
WP_152622273.1       ----------------MAAEETTSAHHVD-----SSVESTPVQAAASASAPEKLDELEEINFLLEEIE-SKIAPLALA---------
WP_191261599.1       ----------------MASEETLSRAAAVESSV-------ASTSQEAAAPAEKLDEIEEIDFLLEEIE-SKIAPLALA---------
WP_169350622.1       ----------------MASEELPSSSVTPAVSAVASPPAEPVAAPVSAAAPEKLDEIEEIDFLLEEIE-SKIAPLALA---------
WP_204221069.1       -------------MAFEETPPTPVVAAEVSTPATQV----ASVHVAAAAAPEKLDELEEINFLLEEIE-SKIAPLALA---------
MBJ6764359.1         ----------------MAAEDTVQSQSEQSTQPVSAEAAPAA----LSAAPEKLDEIEEIDFLLEEIE-SKIAPLALA---------
WP_146210009.1       ----------------MALEETTPVSAEA-----SATESTPSVAAAAPAVPEKLDELEEINFLLEEIE-SKIAPLALA---------
MBZ4418097.1         ----------------MASEENVQSQATPSEQPMSSAPLATASEAAPAAAPEKLDEIEEIDFLLEEIE-SKIAPLALA---------
WP_223755710.1       ---------------------------------MSSAPLATASEAAPAAAPEKLDEIEEIDFLLEEIE-SKIAPLALA---------
WP_143196002.1       ----------------MASEELSASPVASAPAEQQAQAP----E-AVAAAPEKLDELEEIDFLLEEIE-SKIAPLALA---------
MBX7101005.1         ----------------MSAKDDEK-PAAAPAAPEPA-----PAAAPAAPQGEKLEGIEDIDFLLEEIE-NKIAPLALA---------
WP_147422935.1       ----------------MAAEDTVQAQSDQS----TQPTSAEAAPATLTAAPEKLDEIEEIDFLLEEIE-SKIAPLALA---------
WP_157853415.1       ---------------------------------MDNVTVTAETEAEAVDEFDVLDDLDDIEFSLDEIE-NKIAPLALA---------
WP_205525376.1       -----------MASEENVQSQATESEQSMSSSSASLATASAAESNAVSVAPEKLDELEEIDFLLEEIE-SKIAPLALA---------
WP_018731696.1       ----------------------------------MSANRRPTGTRSLDPDVAGLDDLNNVDIDLEKAKIQQPAPLAFTSS-------
WP_147450805.1       -------------------------MPEVAAPATSE----NSAESTDFNELDDIEELDDIEFALDEVE-SSIAPLALAQV-------
WP_224281019.1       --------MERAMED----TAKSTEPEETTEAL-APEAEAAEAETAEDTELDALDELDDVDFDLDEVE-NKIAPLALATTEPSRW--
WP_063346288.1       --------MERAMEDTAKSTAKSTEPEETAEGTEAPQAETAGAETAEDTELDALDELDDVDFDLDEVE-NKIAPLALATTEPSRW—
WP_027660062.1       MEYDMLDNDRSAAPAEVADGTTDNPPLPGDAAPAAPVDSQQASAQPIDPDVEGLDDLDNVDFDLDEVE-NKIAPLALASNEAILWR-
WP_025618787.1       MEYNMLDNDRSVATAEVADGTVDNPPLPVDAATATSRDSQQTSAQPIDPDVEGLDDLDNVDFDLDEVE-NKIAPLALATSEAIQWR-
WP_020678936.1       MENTMVDNDRSAAPAEVADDTAGNPPLPSDASTAASVDSQPAGAQPTDPDVEGLDDLDNVDFDLDEVE-NKIAPLALASNEAIQWR-
ABP55480.1           ----MVDNDRSAAPAEVADDTAGNPPLPSDASTAASVDSQPAGAQPTDPDVEGLDDLDNVDFDLDEVE-NKIAPLALASNEAIQWR-
WP_028564342.1       MENTMVDNDRSAARAEVADDTAGNPPLPSDASTAASVDSQPAGAQPTDPDVEGLDDLDNVDFDLDEVE-NKIAPLALASNEAIQWR-
WP_018223419.1       MEYDMLNNNRSAATADVADGTVGSPPLAASPAAAVSVDSQQTSAQPIDPDVEGLDDLDNVDFDLDEVE-NKIAPLALASNEAIQWR-
WP_018811179.1       MEYDMLNNNRSAATADVADGTVDSPPLAASPAAAVSVDSQQTSAQPIDPDVEGLDDLDNVDFDLDEVE-NKIAPLALASNEAIQWR-
NYT95798.1           MEYDMLDNDRSAATADVADGTVDNPTLAAATATAVAVDSQQSSAQPIDSDVEGLDDLDNVDFDLDEVE-NKIAPLALASNEAIQWR-
WP_019868760.1       MEYDMLDNERSAATAEVADGTVDNLAPAVATTTATSVASQLTDASSIDSDVDGLDDLDNVDFDLDEVE-NKIAPLALASNEAIQWR-
WP_218942224.1       ----MLDNDRSAATADVADGTVDNPTLAAATATAVAVDSQQSSAQPIDSDVEGLDDLDNVDFDLDEVE-NKIAPLALASNEAIQWR-
WP_026184538.1       MEYDMLDNHRSAATADVADGTVDNPTLAAATATAVAVDSQ-SSAQPFGSDVEGLDDLDNVDFDLDEVE-NKIAPLALASNEAIQWR-
WP_018789304.1       MEYAMNDNDRSTAPGEVVDGTVAH------TSASASADSQVTNAQSFDPDVEGLDDLDSVDFDLDEVE-NKIAPLALASNEAILWRP
WP_012183366.1       MEYAMNDNDRSTAPGEVVDGTVAHS--SGDTSASASADSQVTNAQSFDPDVEGLDDLDSVDFDLDEVE-NKIAPLALASNEAILWRP
TQL37960.1           ----MNDNDRSTAPGEVVDGTVAHS--SGDTSASASADSQVTNAQSFDPDVEGLDDLDSVDFDLDEVE-NKIAPLALASNEAILWRP
WP_018908452.1       MEYAMNDNDRSTAPGEVVDGTVAHS--SGDASASASADSQVTNAQSFDPDVEGLDDLDSVDFDLDEVE-NKIAPLALASNEAILWRP
WP_029026774.1       MEYAMNDNDRSAAPEEVIGGTVAHS--SGDTSASASADNQVTNAQSIDPDVEGLDDLDSVDFDLDEVE-NKIAPLALASNEAILWRP
WP_026271378.1       MEYDMLDNARSGATAEVADGTLDNLPPAVATTTATSGNSHLTGAQSIDSDVDGLDDLDNVDFDLDEVE-NKIAPLALASNEAIQWRR
```

Fig. S3. Multiple sequence alignment (MSA) of BhaA homologs. Asp and Glu are highlighted in yellow indicating the high negative charge of these peptides. The highly conserved Ala at the C-terminus is highlighted in red. This Ala is missing in BhaA, but is installed via PEARL-catalyzed addition of Ala in a $tRNA^{Ala}$ dependent reaction by $BhaB_1$.[1, 2] The conserved Pro is highlighted in blue. The alignment was generated using CLUSTAL multiple sequence alignment by Kalign (3.3.1). The last 20 peptides have C-terminal extensions that are likely removed based on a previous study.[1]

```
1        10        20        30        40
MADKVTPEEELDLELEIEDLDDIDFDLEEIEDKVAPLALAW

A40G  -DIDFDLEEIEDKVAPLALGW  A40P  -DIDFDLEEIEDKVAPLALPW
A40V  -DIDFDLEEIEDKVAPLALVW  A40F  -DIDFDLEEIEDKVAPLALFW
A40W  -DIDFDLEEIEDKVAPLALWW  A40D  -DIDFDLEEIEDKVAPLALDW
A40K  -DIDFDLEEIEDKVAPLALKW  A40N  -DIDFDLEEIEDKVAPLALNW
A40S  -DIDFDLEEIEDKVAPLALSW  A40T  -DIDFDLEEIEDKVAPLALTW
```

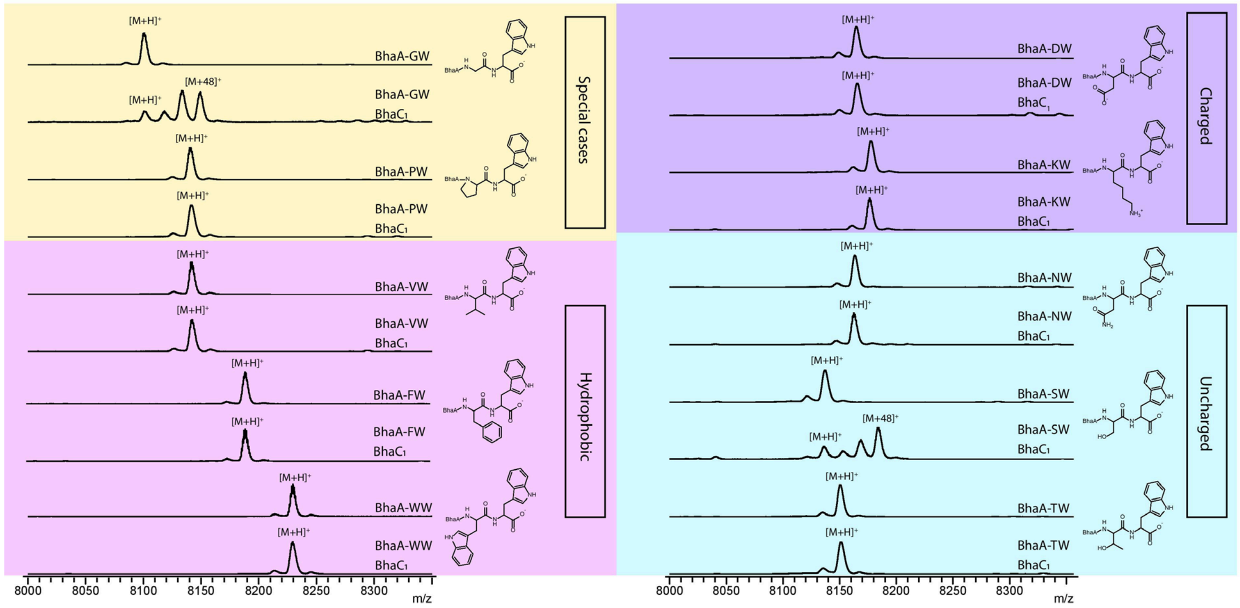

Fig. S4. Evaluation of a BhaA-Xxx-Trp mutant series indicates that the Ala directly N-terminal to the C-terminal Trp is important for BhaC$_1$ modification. MALDI-TOF MS of His$_6$-BhaA-Xxx-Trp mutants expressed with and without BhaC$_1$ in *E. coli.* His$_6$-BhaA-Gly-Trp calculated m/z = 8096.7 Da; observed = 8096.7 Da. His$_6$-BhaA-Gly-Trp* (*denotes [M+ 3 O]$^+$ product) calculated m/z = 8144.7 Da; observed = 8144.5 Da. His$_6$-BhaA-Pro-Trp calculated m/z = 8136.8 Da; observed = 8136.8 Da. His$_6$-BhaA-Val-Trp calculated m/z = 8138.8 Da; observed = 8138.5 Da. His$_6$-BhaA-Phe-Trp calculated m/z = 8186.8 Da; observed = 8185.9 Da. His$_6$-BhaA-Trp-Trp calculated m/z = 8225.8 Da; observed = 8225.6 Da. His$_6$-BhaA-Asp-Trp calculated m/z = 8154.7 Da; observed = 8158.3 Da. His$_6$-BhaA-Lys-Trp calculated m/z = 8167.8 Da; observed = 8171.3 Da. His$_6$-BhaA-Asn-Trp calculated m/z = 8153.8 Da; observed = 8157.4 Da. His$_6$-BhaA-Ser-Trp calculated m/z = 8126.7 Da; observed = 8130.9 Da. His$_6$-BhaA-Ser-Trp* calculated m/z = 8174.7 Da; observed = 8178.1 Da. His$_6$-BhaA-Thr-Trp calculated m/z = 8140.7 Da; observed = 8145.5 Da.

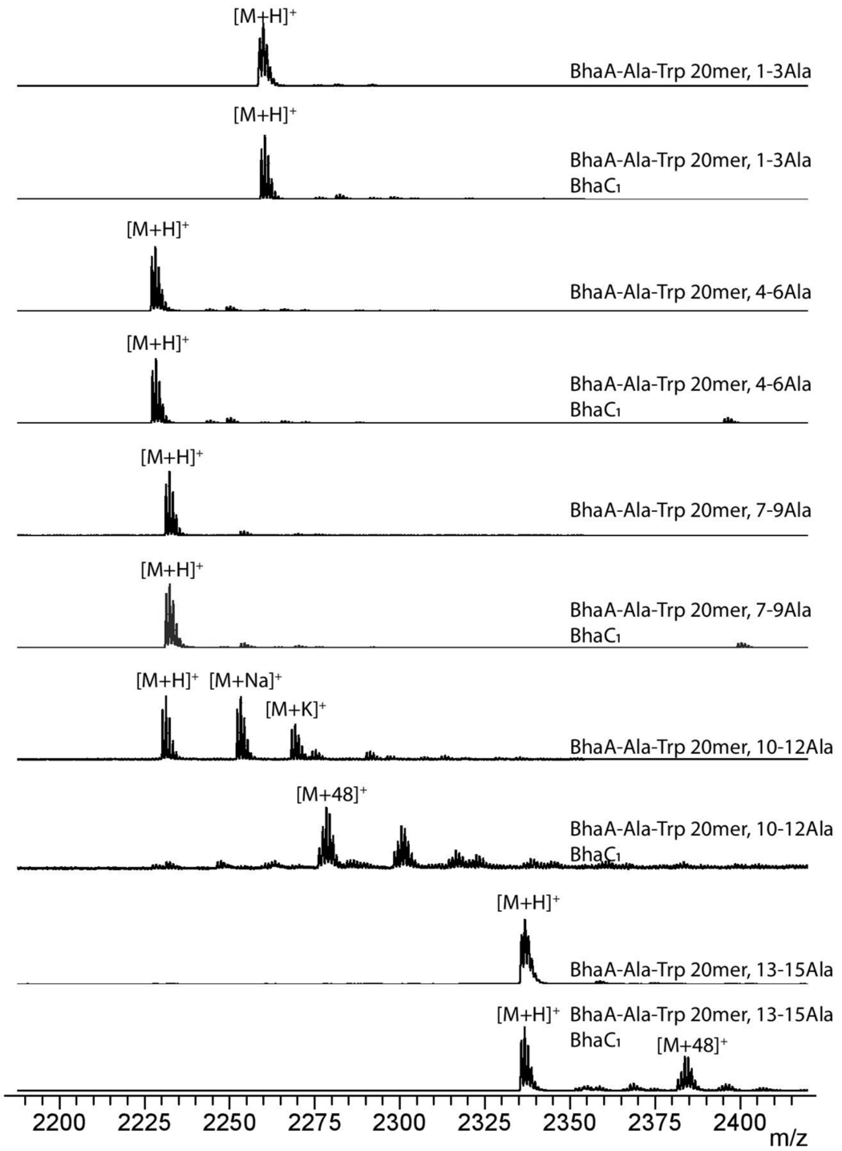

Fig. S5. Use of the MBP-20mer triple-alanine mutant series indicates that the first nine amino acids in the 20mer are critical for $BhaC_1$ modification. MALDI-TOF MS of MBP-20mer alanine mutant series expressed with and without $BhaC_1$. MBP-conjugates were subjected to TEV cleavage then analyzed via MALDI-TOF MS. 1-3Ala calculated m/z = 2259.1 Da; observed = 2259.4 Da. 4-6Ala calculated m/z = 2227.1 Da; observed = 2227.3 Da. 7-9Ala calculated m/z = 2231.1 Da; observed = 2231.3 Da. 10-12Ala calculated m/z = 2230.1 Da; observed = 2230.3, 10-12Ala* (*denotes $[M+3O]^+$ product) calculated m/z = 2278.1 Da; observed = 2278.4 Da. 13-15Ala calculated m/z = 2335.1 Da; observed = 2335.8 Da. 13-15Ala* calculated m/z = 2383.1 Da; observed = 2383.7 Da.

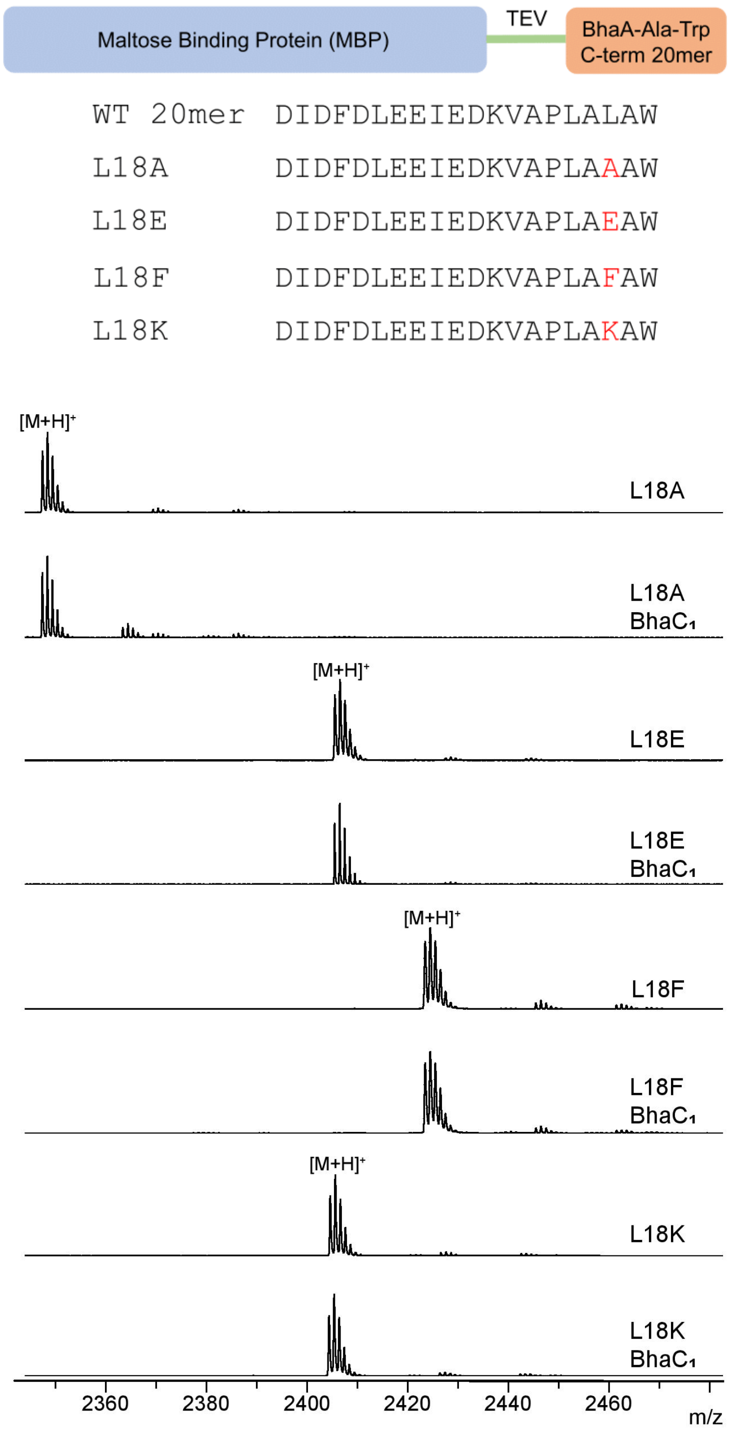

Fig. S6. MBP-20mer Leu18 mutant series indicates Leu18 (Leu39 in BhaA) is crucial for $BhaC_1$ modification. MALDI-TOF MS of MBP-20mer Leu18 mutant series expressed with and without $BhaC_1$ in *E. coli.* MBP-conjugates were subjected to TEV cleavage then analyzed via MALDI-TOF MS. L18A calculated m/z = 2347.1 Da; observed = 2347.4 Da. L18E calculated m/z = 2405.1 Da; observed = 2405.5 Da. L18F calculated m/z = 2423.2 Da; observed = 2423.5 Da. L18K calculated m/z = 2404.2 Da; observed = 2404.2 Da.

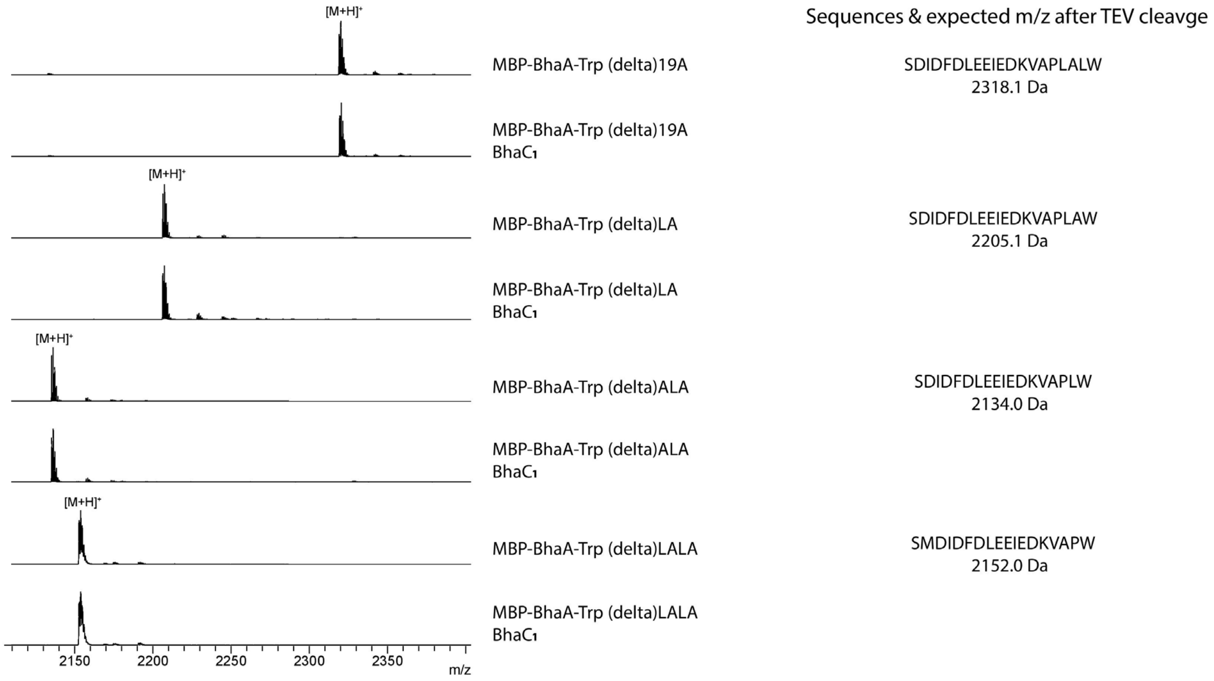

Fig. S7. Deletions in the LALA sequence of the MBP-20mer indicate that the distance between the MN-terminal nine residues (recognition residues) and C-terminal Trp is important. MALDI-TOF MS of MBP-20mer LALA-domain deletions mutant series expressed with and without $BhaC_1$ in *E. coli.* MBP-conjugates were subjected to TEV cleavage then analyzed via MALDI. Δ19 calculated m/z = 2318.1 Da; observed = 2319.0 Da. ΔLA calculated m/z = 2205.1 Da; observed = 2206.3 Da. ΔALA calculated m/z = 2134.0 Da; observed = 2135.2 Da. ΔLALA calculated m/z = 2152.0 Da; observed = 2152.7 Da.

```
BhaC1                                       MLSE------------------RTKLDVEWDRKKIPYTGDVVSHVLKVFDQKKSKDKNCC    42
Desmospora_sp_8437_EGK12454.1               MKGERGRGRMLKSHTLKWKAPKKMDITTLGPSL------------------ELSLQGTGQ    42
Brevibacillus_sp_MCWH_WP_171564579.1        --------MKVKSQTSRLKAQREMDAPTLWKTL------------------KRRLQERGH    34
Anoxybacillus_sediminis_WP_230077042.1      --------MKVKSQTSRLKAQREMDAPTLWKTL------------------KRRLQERGH    34
Paenibacillus_xylanexedens_WP_124114932.1   MRSN------------------EVGDPFLFESKIKSEYEKIELNVLKILELKRGQLPLKI    42
Pseudovibrio_exalbescens_WP_028481737.1     ------------------------------------------------MLQSIQNSQVLT    12
Rhizobium_leguminosarum_WP_129417170.1      ----------------------------------------------------MTQSQVVN    8
Saccharothrix_sp_NRRL_B-16314_WP_033442357.1------------------------------------------MNDADILTATKSAAPVAT    18
Actinoplanes_flavus_WP_208467297.1          ---------------------------------------------------------MSA    3
AmmC1                                       -----------------------------------------------------------M    1
Myxococcus_fulvus_GEN09191.1                ------------------------------------------------------------    0

BhaC1                                       EDKVWVLTH------KKENR---------------IAIIEELKQHLVNNI--ETLTIGGV    79
Desmospora_sp_8437_EGK12454.1               ENMFWFLDA------ENS-L---------------ETLRESL-DLQ--VH--FVVVDGTE    75
Brevibacillus_sp_MCWH_WP_171564579.1        DSMFWLLDV------DHG-I---------------AAVRENLADMD--VK--IFVVDGTE    68
Anoxybacillus_sediminis_WP_230077042.1      DSMFWLLDV------DHG-I---------------AAVRENLADMD--VK--IFVVDGTE    68
Paenibacillus_xylanexedens_WP_124114932.1   DDNIWIYNG------NMLHK---------------AALAEKMHTRLAHRT--NLITIGGT    79
Pseudovibrio_exalbescens_WP_028481737.1     SPQAYFVPV------ALEPD---------------RAEARLLEQVPTAKPLVLRVRPVVG    51
Rhizobium_leguminosarum_WP_129417170.1      DNVAWIETT------ATA-H---------------AAEAAAMA-RGLRFNSVVRIEPDPK    45
Saccharothrix_sp_NRRL_B-16314_WP_033442357.1DAAMVAERVTAICDAAADGRKGPRSAIFLGTVPLLD-LA--REAVRDGRVLDVDCATGG-    74
Actinoplanes_flavus_WP_208467297.1          HTQRWPSRISDLVRTVHEGLPGPRSLVLTSDAGDLTGACRDAAQQSGGMCIEIPCADGG-    62
AmmC1                                       VNRPSDPRISSLLDGARTPLP------------------------GGAGTVRVPVDTGP-    36
Myxococcus_fulvus_GEN09191.1                ---------------------------------------------------------MAL-    3

BhaC1                                       WETTGYSGIATQLRTIITYLL-KKG--YHNFLETFAPEIIYLFPDLTSVPPFRNAKQLDA    136
Desmospora_sp_8437_EGK12454.1               SDSLGFSSLMPLIHDALKVLIDDQG--EAAWLTSVASEVLYLMPNLRDHELFRSAPDLDT    133
Brevibacillus_sp_MCWH_WP_171564579.1        SDTLGFSSLMPLIREALGVLMDDKR--EEAWLKSVASEVLYLMPELRSDEKFRSAPALDA    126
Anoxybacillus_sediminis_WP_230077042.1      SDTLGFSSLMPLIREALGVLMDDKR--EEAWLKSVASEVLYLMPELRSDEKFRSAPALDA    126
Paenibacillus_xylanexedens_WP_124114932.1   WETRGFSSIAPLIYTIISFLL-HKG--EDKLLKNHAPEILYLFPAFSSEPPFVNAQKLED    136
Pseudovibrio_exalbescens_WP_028481737.1     DATLGFGSIAPLVARALSWAEADPE--NKEFLQSIAAELTWLFPELRSKEFFKAAPELTE    109
Rhizobium_leguminosarum_WP_129417170.1      DTLIGFSAVRPAVSQAIVDLEAN-G--HIALLTTLATELVWLFPSLRGRVWTANALDLEA    102
Saccharothrix_sp_NRRL_B-16314_WP_033442357.1--MDSLVPLRSLIEVLVPELRTDAPDLL----DRYSPELVALHPDLADVLDLPVARRLTR    128
Actinoplanes_flavus_WP_208467297.1          --GEPFRPLRGVIERILPIVHAEAIDLT----RQLGAEIVAVHPRLAASFEITPVHTLDE    116
AmmC1                                       --AASYAGLRAALAR----LRAEGAD-FGGLISRHGPEWAELFPA-----DAPGEPPLAE    84
Myxococcus_fulvus_GEN09191.1                --PTAFGGLRKVLKA----CEDAAPDVHRAIAAAHPSEWNRLFPG-----TTQGVPALED    52
                                                    :   :                        *   : *             *

BhaC1                                       IALSPSRRRLHKESEQMFRVTQMVVSLLLKYVEH---T-GKDVVFLFDQIDKMDEHTIRC    192
Desmospora_sp_8437_EGK12454.1               IAVAPSRRRLHKESEQMFRVTQMVVTLLLKYVSR---Q-ERSVVFHFEHLERMDEHTLTC    189
Brevibacillus_sp_MCWH_WP_171564579.1        IAVAPSRRRLHKESEQMFRVTQMVVTLLVKYARR---Q-ERSILFHFEHLERMDEHTLTC    182
Anoxybacillus_sediminis_WP_230077042.1      IAVAPSRRRLHKESEQMFRVTQMVVTLLVKYARR---Q-ERSILFHFEHLERMDEHTLTC    182
Paenibacillus_xylanexedens_WP_124114932.1   IALPPSRRRLHKESEQMFRVTQTIVKVLLDYTKY---LDHTNTIFLFDQIDRMDEHSLRC    193
Pseudovibrio_exalbescens_WP_028481737.1     IAIAPSRRRLHKESEQILRVTVALGRLLRKWLCS---DECEIGIVHAVHVDGIDEQSVRV    166
Rhizobium_leguminosarum_WP_129417170.1      IALPPSRRRLHKESEAVQRVVISLVRLLRAWRAE---AGG-KTLLHFYRLQEMDEHSARL    158
Saccharothrix_sp_NRRL_B-16314_WP_033442357.1LANTPSERRSHRESEHLFRTVNGLGRLLHDLLER----TGAPVVLVWRNLHEADAATLLA    184
Actinoplanes_flavus_WP_208467297.1          IANTPSERRSHRESERSFRIVNGLARLLHGALDQCPSLKSGPIVLSWPSLHTADVGTLLA    176
AmmC1                                       TALAPSERRLHRESEQNYRVLAVAASALCTGAEQ----TGRPLEL--TGVGGTDLASLRG    138
Myxococcus_fulvus_GEN09191.1                LALSPSERRLHRESEQTFWILTVAARTIVETLRA----SGRPLVL--HGAGECDLVSLRA    106
                                             *  **.** *:***            :                .        *  :

BhaC1                                       FTRLSKCIHHCQAVVV--ATF--SEASDNDPRFNFKINTDNQPYISLAENRNRLLHTAHK    248
Desmospora_sp_8437_EGK12454.1               VARLAQCILKRPVTIT--ASV--HDPGVLDERVSSCAGTEQKKFFHLAKIRTDLLNRIAE    245
Brevibacillus_sp_MCWH_WP_171564579.1        IARLAQCLFSLPAAIT--ATV--HEEGVPDVLAASGAGTGHVAFFPVAKIRIDLLNRIAE    238
Anoxybacillus_sediminis_WP_230077042.1      IARLAQCLFSLPAAIT--ATV--HEEGVPDVLAASGAGTGHVAFFPVAKIRIDLLNRIAE    238
Paenibacillus_xylanexedens_WP_124114932.1   FARLSKCIDDLPVVIV--ATV--SEASNLDSRFPMAADSDIRMHVNLAENRNRLLESLYN    249
Pseudovibrio_exalbescens_WP_028481737.1     MRRVADIMPEGGPLIL--FSR--GGEF--KEP---VVEGLT-PWLGQKERRAAMLEGVMQ    216
Rhizobium_leguminosarum_WP_129417170.1      LRRMVEALPGSDLAIL--ASL--GGAP--SEA-APAVEGALREHIDVGAFRSRMLARLRK    211
Saccharothrix_sp_NRRL_B-16314_WP_033442357.1FRRLSRWADQGQTRLVSLATLVPGGIPNSP---PAGLPAAEQACFDWPAHHARLLDLVRE    241
Actinoplanes_flavus_WP_208467297.1          FRRLSRWANQAGSRLVLVASADPHHGPDVP---EPAVPAELADCFSWREQHRRLLTGVLR    233
AmmC1                                       FMRAHEFARTRPGVRILLADPTAVRAAVLP---EADYRA-----ERALCLRRM---GVG-    186
Myxococcus_fulvus_GEN09191.1                VMRAAEWARLDGLDGTLLLTGWRMRRPH-G---AAAFESRRQAYLDSLCDRMRVPHASG-    161
                                            . *                                               :

BhaC1                                       QTSPLVLEPTLTN--DLFHK--KNTIIASEKNTQLMTKEVQTTTTLLESIHK-RKLDNLD    303
Desmospora_sp_8437_EGK12454.1               KTGVVLCTLKD----E----PVRQAEF-AGEKMVAVNAEYEKTLAFHEALQQ-EDVKAIE    295
Brevibacillus_sp_MCWH_WP_171564579.1        KTGAILCSLKG----KAYQAPARERQP-AGEAAAAANAEREKTRAFREALQR-GDVQAIE    292
Anoxybacillus_sediminis_WP_230077042.1      KTGAILCSLKG----KAYQAPARERQP-AGEAAAAANAEREKTRAFREALQR-GDVQAIE    292
Paenibacillus_xylanexedens_WP_124114932.1   QITPTVHEITDWK--NGYES--KFS-YGNSLKLELTNNETQKTTRLLDGMES-GDTEQLD    303
Pseudovibrio_exalbescens_WP_028481737.1     NLKHVVLDQDARSPVSALEIVEI---DGIAN---LGTREKALVEALNTSIEENDQPETLA    270
Rhizobium_leguminosarum_WP_129417170.1      LIRPEIL-QSPANPNGTHDVPVL---RGLPDAFEIATAEARARAAFSAALLSGGDGTHVG    267
Saccharothrix_sp_NRRL_B-16314_WP_033442357.1QSLGTEVAVEFDAGTPAGTTP----------ALGPTPIGEALL------LLS-IDVERGC    284
Actinoplanes_flavus_WP_208467297.1          QSLAETLPIEDAAPSPAERTV----------PAKAEPGDFQLVRRSLEAFAA-GRAEEGS    282
AmmC1                                       -----AAPADLRPVLPTRPEA----------GPGLGDGGTADARLYADAFGD-GTAFDRL    230
Myxococcus_fulvus_GEN09191.1                -----PGPVSSRELEPTVDLE-----------------GRY-LRLVVD--ES-ESRETRV    195

BhaC1                                       SEVIDALEESIFTQNYEHALFLINKTSPYMDHLK--------------------------    337
Desmospora_sp_8437_EGK12454.1               ETVLQLLEVSVFTKNHDHSLYTIAQVWPILPRFT--------------------------    329
Brevibacillus_sp_MCWH_WP_171564579.1        TAVLELLEVSVFTKNHDHSLRAIAQVWPMLPRFS--------------------------    326
Anoxybacillus_sediminis_WP_230077042.1      TAVLELLEVSVFTKNHDHSLRAIAQVWPMLPRFS--------------------------    326
Paenibacillus_xylanexedens_WP_124114932.1   EILLEALETSVFTQNYEHALFLVNKVSRHIHDLK--------------------------    337
Pseudovibrio_exalbescens_WP_028481737.1     AHAISAIEEAVFTLNFPQARAILGQIGGRLVEFS--------------------------    304
Rhizobium_leguminosarum_WP_129417170.1      RLAAQAVAASVFTLNLPLAIATVAELGDKFSHIA--------------------------    301
Saccharothrix_sp_NRRL_B-16314_WP_033442357.1AQAIRAMGAAAFTLNYEAVLQLAAHVIAAVTGQEEPFDEARFAAEWEAAAPEEYYAAIEF    344
Actinoplanes_flavus_WP_208467297.1          ALAMRAMAPAAFALDYATVLLLCAHVVA-VADNGEPFDQGAFDAAWQASAEAAHHPALEF    341
AmmC1                                       AAVLAGCRRGFFTGNWEAMAALAATGGTLLDGFPDSRVADLLAA---AREQDQQAEAIEF    287
Myxococcus_fulvus_GEN09191.1                AAAILAIRSCFFTTNYEGALLAAEHGLSLLESTSEPNFPGRVVQAWEALDTGFTTPAIEI    255
                                                       *: :              .
```

```
BhaC1                                             --------NKTKVEVWIYIGIGYAFMLKYEKALTLFQY-ALKNSEDTLQKSEIQLLIALL     388
Desmospora_sp_8437_EGK12454.1                     --------VKNRVEVIHLLGILYAYMGDFSIAIQAFKH-AGQFVQEPVQAAENKFFLALL     380
Brevibacillus_sp_MCWH_WP_171564579.1              --------TKNRVEVIHLLGILYAYMGDFPTAIDVFTY-GGQFVSDPVQAAENKFFLALL     377
Anoxybacillus_sediminis_WP_230077042.1            --------TKNRVEVIHLLGILYAYMGDFPTAIDVFTY-GGQFVSDPVQAAENKFFLALL     377
Paenibacillus_xylanexedens_WP_124114932.1         --------KQTIVELWIYMGLCYAYMVEYDKALNLFKH-ALTFTEDKLKKSELHLYIALL     388
Pseudovibrio_exalbescens_WP_028481737.1           --------PKQQHELVLHLAFAEAFACRFESAAEALQY-AMSLADRADEKAMSHFFLALL     355
Rhizobium_leguminosarum_WP_129417170.1            --------AADRHEIALQVGFAHAFSTSFESARQALEV-SAEFAETTEQRAATHFYLALV     352
Saccharothrix_sp_NRRL_B-16314_WP_033442357.1      AVIRPADVRDVLAVAWRAAGFANSCLDDHETSLACYRQ-ALGLADTPLQRARARMYLGLI     403
Actinoplanes_flavus_WP_208467297.1                AVQQPHTREELAGAAWRAAGFANSCLGSHETALRCYHQ-AARLARGHQQQAQALMYLGLI     400
AmmC1                                             EPGILRTTDDVRAFLAKVLGVQATFRGDQDRALAHFRAMRAGERLSPEVRAQSHLYAALT     347
Myxococcus_fulvus_GEN09191.1                      DRASLGDEDELKALLHRCMGVVHVFTGSHDEAMAAFGR-GLECRLPPELRARLHMFRALT     314
                                                                     ..          :                  :   :  .*

BhaC1                                             YTKRLNNPLLGREIIDNALQNIGSLTGN--RVEVERTWLYNLKALTFVERRDLVNAYKNC     446
Desmospora_sp_8437_EGK12454.1                     YTKRLNDSVTGRDSIAQALECMNDVQGE--LADIERAWLNNLCALTYVNEKNRRAAYDCC     438
Brevibacillus_sp_MCWH_WP_171564579.1              YTKRLNDSVTGRDYIAQALECLAGVQGE--LADIERTWLNNLCALTYVNEKNRSAAYECC     435
Anoxybacillus_sediminis_WP_230077042.1            YTKRLNDSVTGRDYIAQALECLAGVQGE--LADIERTWLNNLCALTYVNEKNRSAAYECC     435
Paenibacillus_xylanexedens_WP_124114932.1         CTKRLNTPLVGRGFIDQALQNIADTTGS--EVEVERTWLHNLRALTYVETGDLAQAFKEC     446
Pseudovibrio_exalbescens_WP_028481737.1           KIKRLGQPLQGREHLDHAIAMLKDRTDD--AALNDLAWLYNLRALSFVELRDVANARKQV     413
Rhizobium_leguminosarum_WP_129417170.1            HIKRLGNPVEGRQHVDVALDLLEAAGDA--AHDNEIAWLYNLKALSFVEEKNLAAAGRCL     410
Saccharothrix_sp_NRRL_B-16314_WP_033442357.1      TGKRLRRIAEAEQHLDQGLAEIDGRDDE--DARLERCWLLNVRALMAFQQRDHRRAMLMV     461
Actinoplanes_flavus_WP_208467297.1                TGKRLHRIDEAEAHIRAGIAEVTGDDVDV-VGVLERCWLLNVNALMAYSGGRFKDAMRMA     459
AmmC1                                             LTKRLGSVDEAVAEVEEGFTAVPHREGEGNGVRRERGWLHNLRALTHFARRELREAFGHE     407
Myxococcus_fulvus_GEN09191.1                      LTKRFGQLPNARAEVEAGLAELARSTAP--DRALQEGWLRNVCALTWFQERKLDKALVEE     372
                                                    **:     .   :  .:  :            :  ** *: **          *

BhaC1                                             RKALEHIKKGDRSEDAIHIKINLLSNISVLYEYMNKVDASLTKWMKFDKFILQSSPVFTK     506
Desmospora_sp_8437_EGK12454.1                     LQALKYIKNGHRSSDAIHIKINLISNITVLHEYGKEMNKAVNKWMFFERFIEASGPVFAM     498
Brevibacillus_sp_MCWH_WP_171564579.1              LKALDYIKHGHRSSDAIHIKINLISNITVLHEYGKELDKAIAKWMFFEKFLKASGPVFAK     495
Anoxybacillus_sediminis_WP_230077042.1            LKALDYIKHGHRSSDAIHIKINLISNITVLHEYGKELDKAIAKWMFFEKFLKASGPVFAK     495
Paenibacillus_xylanexedens_WP_124114932.1         KQGLEHIKEGNRKEDAIHIKINLISNISVLFEYMKKVDTALKHWSKFEKFVTHSSPVFTK     506
Pseudovibrio_exalbescens_WP_028481737.1           LMGIKYNKACKRTPDSIHLKINLLSNMTNLEEFTRQVPKAIERWSVFEPMLKSAAGSFAK     473
Rhizobium_leguminosarum_WP_129417170.1            HLAVTRNARPARSSDQVHLKLNLLNNITLLEEISGRYDQAIARYAMLEPLMRTASPSAAK     470
Saccharothrix_sp_NRRL_B-16314_WP_033442357.1      REAREVMR-PLHSSEATHLKINLISNISVLLEKTGRTDKAVALWQQFAAFLGPSNELFAK     520
Actinoplanes_flavus_WP_208467297.1                REAWATVR-PIQSSEATHLKVNLVSNISVLYEKAGQPAEAVKVWEKFRALLGRANALFAK     518
AmmC1                                             KQALACIE-GLDDPSSIHLRVNLFSNVSVLQEKAGRHQQAARTWSRFKEAEGSANATFVK     466
Myxococcus_fulvus_GEN09191.1                      KLAMRCVG-DLHDASATHLKINLISNASYLQESARQFADAIGTWRRFEGISERWGVNFSK     431
                                                    .           .  *:::**..* : * *   .   :   :  :

BhaC1                                             HYLYRKAGLLYKMKNYLEAIEALKASYKIAAELHDYFHMDIIARGLGAILFEEENFSEAI     566
Desmospora_sp_8437_EGK12454.1                     HYHYRKGGLQYRNRQVVDAIESLMDSYRLAVDLHSSFYQDVIARGLAGIYFMEEQYEQAA     558
Brevibacillus_sp_MCWH_WP_171564579.1              HYYYRKGGLQYKNRQVAEAITSLTASYRLACDLHSLFYQDVIARGLAGVYFWEGQYEEAA     555
Anoxybacillus_sediminis_WP_230077042.1            HYYYRKGGLQYKNRQVAEAITSLTASYRLAWDLHSLFYQDVIARGLAGVYFWEGQYEEAA     555
Paenibacillus_xylanexedens_WP_124114932.1         HYLYRKAGLLFKLQRYAEAIQSLKDSYRIAKDMNDAFHMDIIARSLGAIYFEQRLYDKAT     566
Pseudovibrio_exalbescens_WP_028481737.1           HYHYRMGGLLTKVDRFDEASDHFMKAYAEARKSHDLFYAVLIARDLGGVLYRAGQVEEAA     533
Rhizobium_leguminosarum_WP_129417170.1            HWHYRLGALMVRCGRREEGIVHLEAAHASAERDEDLYYCDRIAADIAAVQEACGDRTNAT     530
Saccharothrix_sp_NRRL_B-16314_WP_033442357.1      HYYFREGGLRLLAGRSEDALTSYRNSYEQCVRIDDPFHAAVVARAAGYVAHQLGRQDEAV     580
Actinoplanes_flavus_WP_208467297.1                HYHFREAGLRLAAGQLDEALEAYQASFDQCEATTDPFHAQIVAEACAHVEFKKGAVAEAI     578
AmmC1                                             HHAYRAGGLALLCGDRDTAREDLAKSIACAAEFNDDFHEAEIRTEVGTLLLADGDRAGAA     526
Myxococcus_fulvus_GEN09191.1                      HHRYRLAGLEFASGQRDEAVEHFQQAYASAEALRDSFHRQVIAAELGRLFLDDGRMAQAV     491
                                                  *  :* ..*         .      :   .    . ::   :    . :         *

BhaC1                                             QWFRCSLNAKYKLLEDEDIPRVVAAIAICFNQLGESHKGMKEIEACLQT----HTKGDAA     622
Desmospora_sp_8437_EGK12454.1                     RWYQLATDAKKTLLQEDDLPKVALGHALSLYKLGRGEEGEKVLRDALLF----QPAGDAA     614
Brevibacillus_sp_MCWH_WP_171564579.1              HWYQLAIDAKKKLLQEDNLPKVALGRALSLRKQGRTLEAEQVLREALDL----QPTGDAA     611
Anoxybacillus_sediminis_WP_230077042.1            HWYQLAIDAKKKLLQEDNLPKVALGRALSLRKQGRTLEAEQVLREALDL----QPTGDAA     611
Paenibacillus_xylanexedens_WP_124114932.1         QWYRRSLDSKLLLIQDEDLPRVATALAICLEKQGNGDEGKQEILNSLKI----QSKGSAA     622
Pseudovibrio_exalbescens_WP_028481737.1           RWFEACVRLQDEMGADDAISADWVALWYCRWCVTSKTAGANAVKQPLYKSISSGSMGHEG     593
Rhizobium_leguminosarum_WP_129417170.1            LWYRRAAAIEREIGNATGLDHVNAAL----------------ARMALLPAMDDGKIE---     571
Saccharothrix_sp_NRRL_B-16314_WP_033442357.1      EWYERVVRAGEACGDHEALTADRGTLAF--------------------------------     608
Actinoplanes_flavus_WP_208467297.1                SWYERAVDAAWTVGDHERLPSMQATLAG--------------------------------     606
AmmC1                                             AHFERAVALTSRIGDPYRMALAQLGRARAGGADPDP-----SVAALARLS---TTHPARG     578
Myxococcus_fulvus_GEN09191.1                      EWFARAEQHAREIGEPLKAAESLAGLSLAAGREDWS-----EALRCARAS---TTWPKET     543
                                                    :

BhaC1                                             QMVLDMLTQWEHQDNDKWKSYVKWAIQKPDSKLNRPFDLTNLYCERTESS---            672
Desmospora_sp_8437_EGK12454.1                     EKATEALSRWHDVPSEELEALIQYAIEKPDTKLNRPFDLTNLY----------            657
Brevibacillus_sp_MCWH_WP_171564579.1              EKAKKALSRWNDLSAEELEELISYAIDKPDTKLNRPFDLTNLY----------            654
Anoxybacillus_sediminis_WP_230077042.1            EKAKKALSRWNDLSAEELEELISYAIDKPDTKLNRPFDLTNLY----------            654
Paenibacillus_xylanexedens_WP_124114932.1         EKASEILLRWDQKKDETWESYVTWSIQKPETKLNRPFDLTNLYSEKPERIRVG            675
Pseudovibrio_exalbescens_WP_028481737.1           E---------DQSGANAFAPVEFQFLPRPGTKLNRPFDITNLYWESAS-----            632
Rhizobium_leguminosarum_WP_129417170.1            -------------DDRRAAPESFRPLSAIKTRLNQPFHQINLY----------            601
Saccharothrix_sp_NRRL_B-16314_WP_033442357.1      --LS---------------QDGGSAPVRPDTKLNRPFSLVNTYLAGEG-----            639
Actinoplanes_flavus_WP_208467297.1                --LV---------------DGSGSLDAVPPTKLNRPFDLVNVAN---------            633
AmmC1                                             AALADAAE-RG-------GDAADGLLPAVRTKLNRPFDLINFQE---------            614
Myxococcus_fulvus_GEN09191.1                      QALVDALTKSD-------AKAVHALLPRPRTKLNRPFDSVSLY----------            579
                                                                                ::**:**   .
```

Fig. S8. Multiple sequence alignment (MSA) of $BhaC_1$ homologs. Homologs were identified by BlastP and were selected to highlight diversity of organisms; these are not the proteins that are most identical to $BhaC_1$. The alignment was generated using CLUSTAL O (1.2.4). Residues that line the cleft/tunnel and coordinate the substrate peptides are highlighted in yellow. A subset of these residues are highly conserved (*) or are substituted by similar amino acids (:) in homologs.

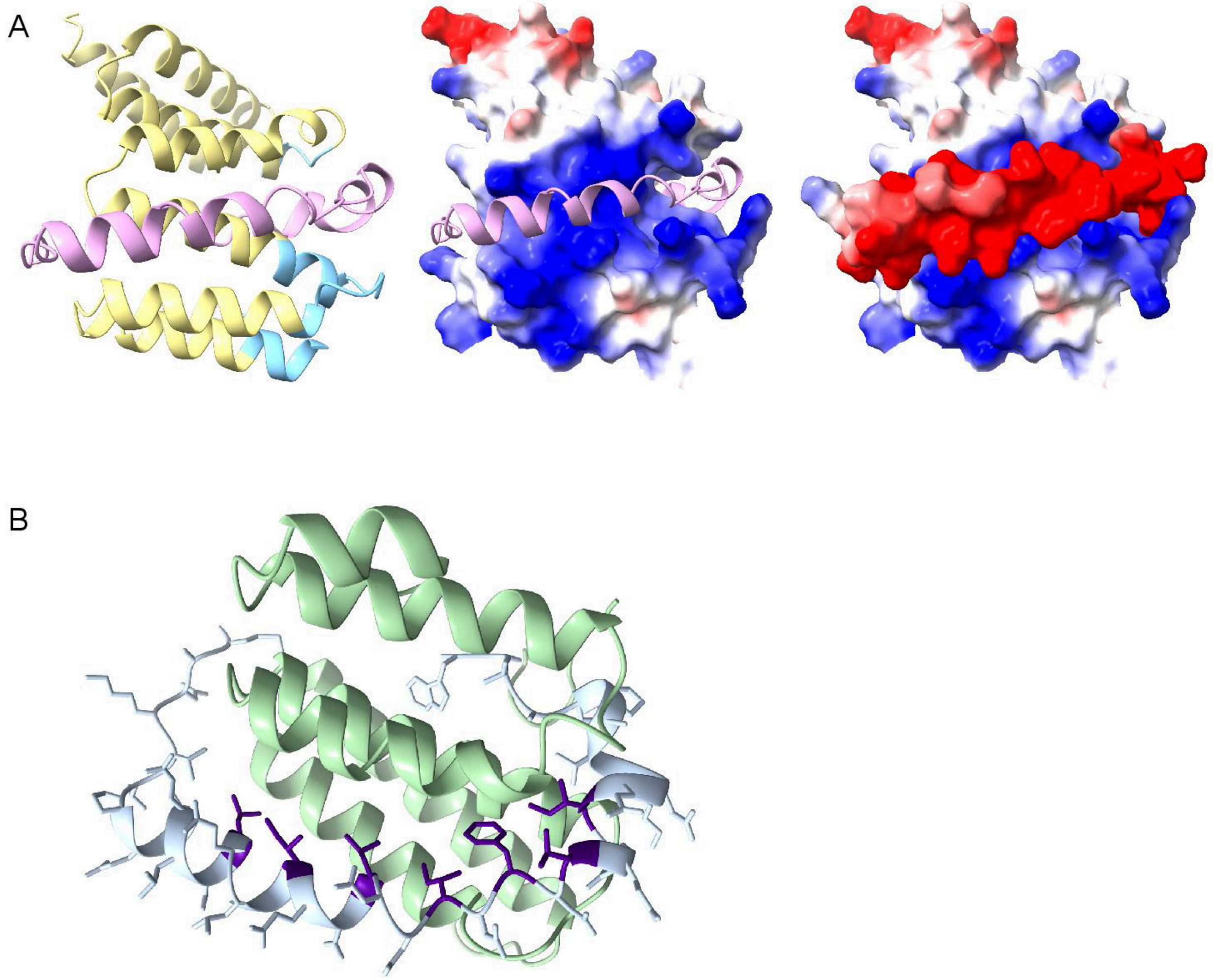

Fig. S9. AlphaFold multimer analysis of BhaA-Ala-Trp bound to BhaC$_1$. (A) Residues Asp19 – Asp35 of BhaA form an alpha-helix that is predicted to interact electrostatically with the positively charged cleft in the TPR domain of BhaC$_1$. In the ribbon model: Blue – BhaC$_1$, Yellow – TPR domain, Pink: BhaA-Ala-Trp. Middle and right panels are colored according to electrostatics (red negatively charged, blue positively charged, white hydrophobic). (B) Ribbon diagram showing the stretch of hydrophobic residues of BhaA-Ala-Trp in the α-helix and turn that interact with BhaC$_1$. BhaA-Ala-Trp shown in grey, hydrophobic residues shown in purple. BhaC$_1$ shown in green.

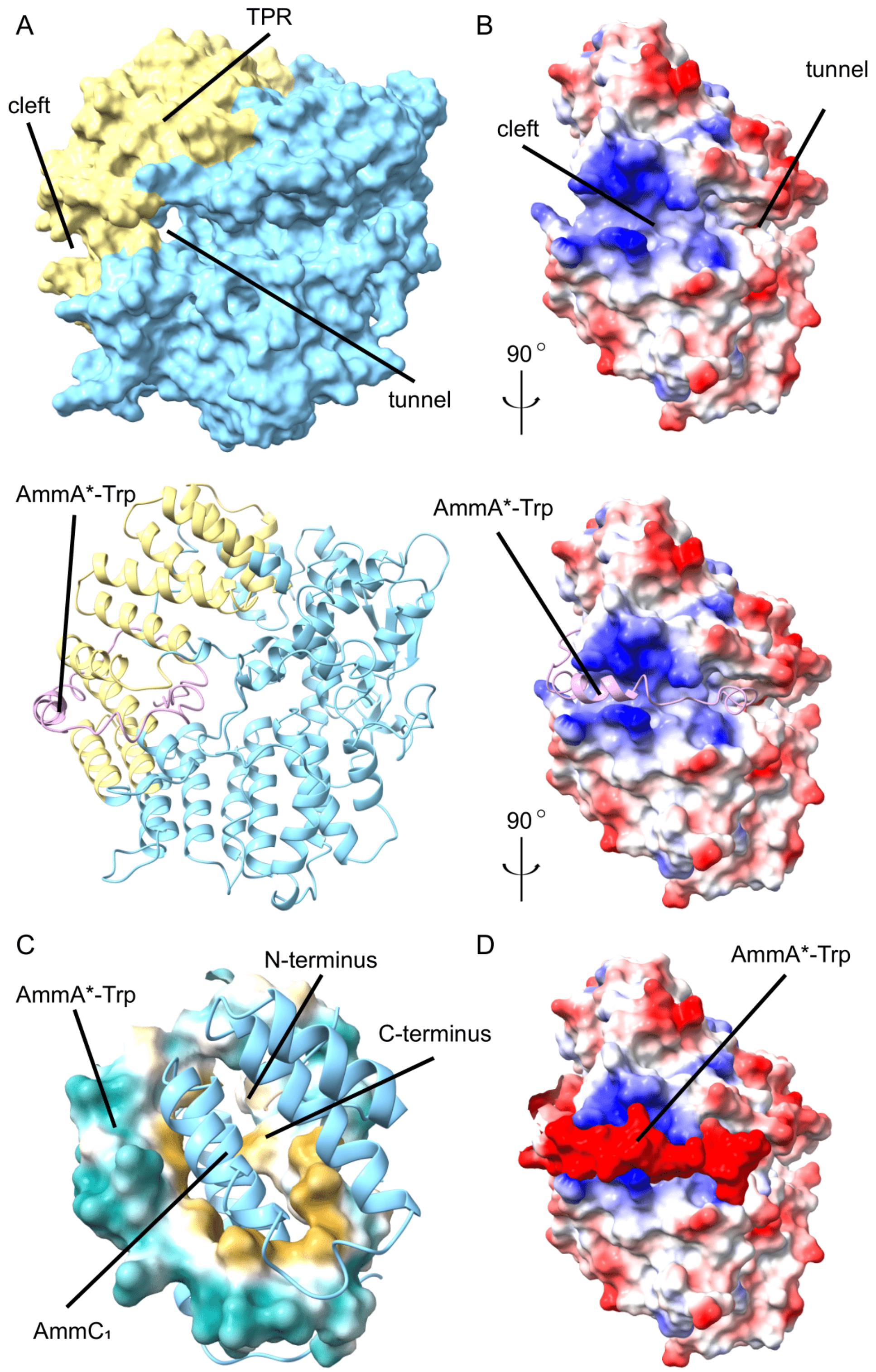

Fig. S10. AlphaFold analysis of $AmmC_1$ and its substrate, AmmA*-Trp. The AlphaFold analysis was performed with a His_6-tag (GSSHHHHHHSQDP) on AmmA*-Trp. (A) $AmmC_1$ is shown in blue, the TPR domain is shown in yellow, AmmA*-Trp is shown in pink. The AlphaFold prediction features a tunnel and the Multimer algorithm predicts that the C-terminus of AmmA*-Trp will bind in this tunnel.

(B) $AmmC_1$ space-filling structure showcasing electrostatics. The positively charged cleft in the TPR domain coordinates the helical and negatively charged N-terminal portion of AmmA*-Trp. (C) Hydrophobicity plot using the Kyte-Doolittle scale made in ChimeraX.[3] The face of the AmmA*-Trp α-helix and subsequent turn that faces $AmmC_1$ is made up of hydrophobic amino acids. Yellow indicates hydrophobic, teal indicates hydrophilic and white indicates neutral. $AmmC_1$ is shown in blue. (D) The negatively charged amino acids on AmmA*-Trp (red) face outwards but make extensive interactions with the positively charged side chains of Lys and Arg residues on $AmmC_1$ (blue; for specific interactions see Figure S13).

```
BhaA-Ala-Trp      -----MAD----KVTPEEELDLELEIEDLDDIDFDLEEIEDKVAPLALAW        41
AmmA*-Trp         MSETQVTETDNPAEEPAEIAAESDDLADLDDIEFDLDEVESKIAPLALAW        50
                       :::       * *    . :: *****:***:*:*.*:*******
```

Fig. S11. Pairwise sequence alignment of BhaA-Ala-Trp and AmmA*-Trp. Alignment was generated using Clustal O (1.2.4).

```
BhaC1      MLSERTKLDVEWDRKKIPYTGDVVSHVLKVFDQKKSKDKNCCEDKVWVLTHKKENRIAI-     59
AmmC1      -----------------------------------------------MVNRPSDPRISSL     13
                                                          ::.: .: **:

BhaC1      IEELKQHLVNNIETLTIG-GVWETTGYSGIATQLRTIITYLLKKGYHNFLETFAPEIIYL     118
AmmC1      LDGARTPLPGGAGTVRVPVDTGPAASYAGLRAALARLR--AEGADFGGLISRHGPEWAEL     71
           ::  :  * ..  *: :  ..  ::.*:*: : *  :       .: .::. ..**   *

BhaC1      FPDLTSVPPFRNAKQLDAIALSPSRRRLHKESEQMFRVTQMVVSLLLKYVEHTGKDVVFL     178
AmmC1      FPADAPG-----EPPLAETALAPSERRLHRESEQNYRVLAVAASALCTGAEQTGRPLELT     126
           **  :          *   **:**.****:**** :**  :..* * . .*:**: : :

BhaC1      FDQIDKMDEHTIRCFTRLSKCIHHCQAVVVATF---SEASDNDPRFNFKINTDNQPYISL     235
AmmC1      --GVGGTDLASLRGFMRAHEFARTRPGVRILLADPTAVRAAVLPEADYR-----------     173
             :.  *  ::* * *  :  :   .* :      :  :   *. :::

BhaC1      AENRNRLLHTAHKQTSPLVLEPTLTNDLFHKKNTIIASEKNTQLMTKEVQTTTTLLESIH     295
AmmC1      -AERALCLRRMGVGAAPADLRPVLPTRPEAGPGLGDGGTADARLYADAFGDGT-------     225
             :*   *:     ::*  *.*.* .       .   ..  :::* :. .   *

BhaC1      KRKLDNLDSEVIDALEESIFTQNYEHALFLINKTSPYMDH--------------------     335
AmmC1      --AFDRL-AAVLAGCRRGFFTGNWEAMAALAATGGTLLDGFPDSRVADLLAAAREQDQQA     282
             :*.* : *: . ...:** *:*    *  .  .  :*

BhaC1      ---------LKNKTKVEVWI--YIGIGYAFMLKYEKALTLFQYALKNSE-DTLQKSEIQL     383
AmmC1      EAIEFEPGILRTTDDVRAFLAKVLGVQATFRGDQDRALAHFRAMRAGERLSPEVRAQSHL     342
                    *:.. .*..::   :*:  :*  . ::**: *:    ... .   ::: :*

BhaC1      LIALLYTKRLNNPLLGREIIDNALQN--IGSLTGNRVEVERTWLYNLKALTFVERRDLVN     441
AmmC1      YAALTLTKRLGSVDEAVAEVEEGFTAVPHREGEGNGVRRERGWLHNLRALTHFARRELRE     402
             **  ****..   .   :::.:      .  ** *. ** **:**:***.. **:* :

BhaC1      AYKNCRKALEHIKKGDRSEDAIHIKINLLSNISVLYEYMNKVDASLTKWMKFDKFILQSS     501
AmmC1      AFGHEKQALACIE-GLDDPSSIHLRVNLFSNVSVLQEKAGRHQQAARTWSRFKEAEGSAN     461
           *: : ::**  *: *  . .:**:::**:**:*** *  .: : :  .* :*.:   .:.

BhaC1      PVFTKHYLYRKAGLLYKMKNYLEAIEALKASYKIAAELHDYFHMDIIARGLGAILFEEEN     561
AmmC1      ATFVKHHAYRAGGLALLCGDRDTAREDLAKSIACAAEFNDDFHEAEIRTEVGTLLLADGD     521
            .*.**: ** .**     :   * * *  *   ***::* **   *   :*::*: : :

BhaC1      FSEAIQWFRCSLNAKYKLLEDEDIPRVVAAIAICFNQLGESHKGMKEIEACLQTHTK---     618
AmmC1      RAGAAAHFERAVALTSR---------IGDPYRMALAQLGRARAGGADPDPSVAALARLST     572
            : *   *. ::  . :         :     :.: ***.:: *  : : .: : ::

BhaC1      -GDAAQMVLDMLTQWEHQDNDKWKSYVKWAIQKPDSKLNRPFDLTNLYCERTESS  672
AmmC1      THPARGAAL---ADAAERG----GDAADGLLPAVRTKLNRPFDLINFQE------  614
```

Fig. S12. Pairwise sequence alignment of $BhaC_1$ and $AmmC_1$. Alignment was generated using Clustal O (1.2.4). Conserved residues that line the cleft/tunnel and coordinate the substrate peptides are highlighted in yellow.

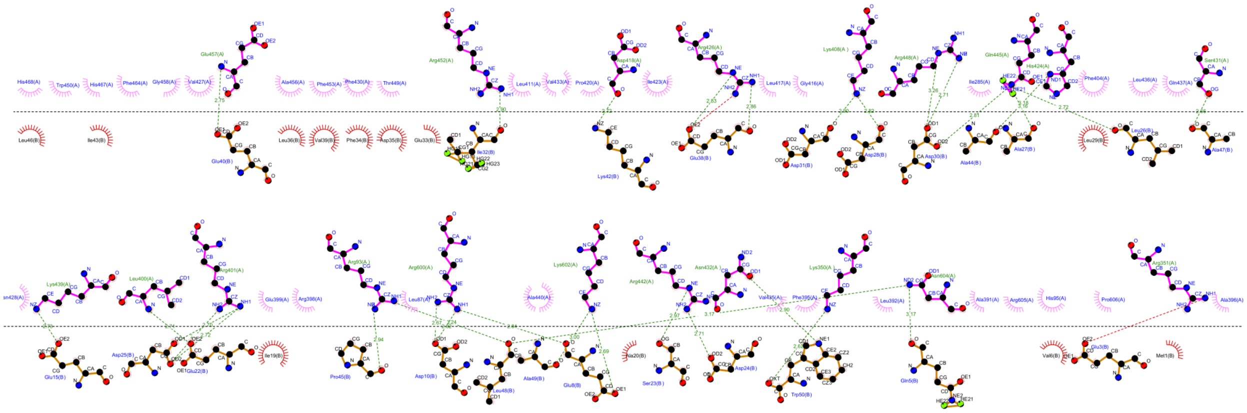

Fig. S13. DimPlot showcasing interactions between AmmC$_1$ and AmmA*-Trp. AmmC$_1$ is the sequence for which the residues are denoted with labels (A), AmmA*-Trp is the sequence for which the residues are denoted with labels (B). AmmA*-Trp was submitted as the full-length sequence to DimPlot analysis (Met1-Trp50).

Table S1. Oligonucleotide (gBlock) sequences for His$_6$-BhaA-Xxx-Trp mutants and BhaA-Ala-Trp-Gly for construction of pET28b-His$_6$-BhaA-Xxx-Trp or pET28b-His$_6$-BhaA-Ala-Trp-Gly

BhaA-AW

TTGCTCAGCGGTGGCAGCAGCCAACTCAGCTTCCTTTCGGGCTTTGTTAGCAGCCGGATCTCAGTGGTGG
TGGTGGTGGTGCTCGAGTGCGGCCGCAAGCTTGTCGACGGAGCTCGAATTCGGATTACCAGGCAAGAGCC
AGAGGAGCGACTTTATCTTCAATTTCCTCTAGATCAAAGTCGATGTCATCAAGGTCTTCAATTTCTAATT
CTAGATCTAACTCTTCTTCAGGTGTAACCTTGTCGGCCATTCCCGACCCATTTGCTGTCCACCAGTCATG
CTAGCCATATGGCTGCCGCGCGGCACCAGGCCGCTGCTGTGATGATGATGATGATGGCTGCTGCCCATGG
TA

BhaA-KW

TTGCTCAGCGGTGGCAGCAGCCAACTCAGCTTCCTTTCGGGCTTTGTTAGCAGCCGGATCTCAGTGGTGG
TGGTGGTGGTGCTCGAGTGCGGCCGCAAGCTTGTCGACGGAGCTCGAATTCGGATTACCATTTAAGAGCC
AGAGGAGCGACTTTATCTTCAATTTCCTCTAGATCAAAGTCGATGTCATCAAGGTCTTCAATTTCTAATT
CTAGATCTAACTCTTCTTCAGGTGTAACCTTGTCGGCCATTCCCGACCCATTTGCTGTCCACCAGTCATG
CTAGCCATATGGCTGCCGCGCGGCACCAGGCCGCTGCTGTGATGATGATGATGATGGCTGCTGCCCATGG
TA

BhaA-DW

TTGCTCAGCGGTGGCAGCAGCCAACTCAGCTTCCTTTCGGGCTTTGTTAGCAGCCGGATCTCAGTGGTGG
TGGTGGTGGTGCTCGAGTGCGGCCGCAAGCTTGTCGACGGAGCTCGAATTCGGATTACCAATCAAGAGCC
AGAGGAGCGACTTTATCTTCAATTTCCTCTAGATCAAAGTCGATGTCATCAAGGTCTTCAATTTCTAATT
CTAGATCTAACTCTTCTTCAGGTGTAACCTTGTCGGCCATTCCCGACCCATTTGCTGTCCACCAGTCATG
CTAGCCATATGGCTGCCGCGCGGCACCAGGCCGCTGCTGTGATGATGATGATGATGGCTGCTGCCCATGG
TA

BhaA-VW

TTGCTCAGCGGTGGCAGCAGCCAACTCAGCTTCCTTTCGGGCTTTGTTAGCAGCCGGATCTCAGTGGTGG
TGGTGGTGGTGCTCGAGTGCGGCCGCAAGCTTGTCGACGGAGCTCGAATTCGGATTACCACACAAGAGCC
AGAGGAGCGACTTTATCTTCAATTTCCTCTAGATCAAAGTCGATGTCATCAAGGTCTTCAATTTCTAATT
CTAGATCTAACTCTTCTTCAGGTGTAACCTTGTCGGCCATTCCCGACCCATTTGCTGTCCACCAGTCATG
CTAGCCATATGGCTGCCGCGCGGCACCAGGCCGCTGCTGTGATGATGATGATGATGGCTGCTGCCCATGG
TA

BhaA-FW

TTGCTCAGCGGTGGCAGCAGCCAACTCAGCTTCCTTTCGGGCTTTGTTAGCAGCCGGATCTCAGTGGTGG
TGGTGGTGGTGCTCGAGTGCGGCCGCAAGCTTGTCGACGGAGCTCGAATTCGGATTACCAAAAAAGAGCC
AGAGGAGCGACTTTATCTTCAATTTCCTCTAGATCAAAGTCGATGTCATCAAGGTCTTCAATTTCTAATT
CTAGATCTAACTCTTCTTCAGGTGTAACCTTGTCGGCCATTCCCGACCCATTTGCTGTCCACCAGTCATG
CTAGCCATATGGCTGCCGCGCGGCACCAGGCCGCTGCTGTGATGATGATGATGATGGCTGCTGCCCATGG
TA

BhaA-WW

TTGCTCAGCGGTGGCAGCAGCCAACTCAGCTTCCTTTCGGGCTTTGTTAGCAGCCGGATCTCAGTGGTGG
TGGTGGTGGTGCTCGAGTGCGGCCGCAAGCTTGTCGACGGAGCTCGAATTCGGATTACCACCAAAGAGCC
AGAGGAGCGACTTTATCTTCAATTTCCTCTAGATCAAAGTCGATGTCATCAAGGTCTTCAATTTCTAATT
CTAGATCTAACTCTTCTTCAGGTGTAACCTTGTCGGCCATTCCCGACCCATTTGCTGTCCACCAGTCATG
CTAGCCATATGGCTGCCGCGCGGCACCAGGCCGCTGCTGTGATGATGATGATGATGGCTGCTGCCCATGG
TA

BhaA-GW

TTGCTCAGCGGTGGCAGCAGCCAACTCAGCTTCCTTTCGGGCTTTGTTAGCAGCCGGATCTCAGTGGTGG
TGGTGGTGGTGCTCGAGTGCGGCCGCAAGCTTGTCGACGGAGCTCGAATTCGGATTACCAGCCAAGAGCC
AGAGGAGCGACTTTATCTTCAATTTCCTCTAGATCAAAGTCGATGTCATCAAGGTCTTCAATTTCTAATT
CTAGATCTAACTCTTCTTCAGGTGTAACCTTGTCGGCCATTCCCGACCCATTTGCTGTCCACCAGTCATG
CTAGCCATATGGCTGCCGCGCGGCACCAGGCCGCTGCTGTGATGATGATGATGATGGCTGCTGCCCATGG
TA

BhaA-PW

TTGCTCAGCGGTGGCAGCAGCCAACTCAGCTTCCTTTCGGGCTTTGTTAGCAGCCGGATCTCAGTGGTGG
TGGTGGTGGTGCTCGAGTGCGGCCGCAAGCTTGTCGACGGAGCTCGAATTCGGATTACCACGGAAGAGCC
AGAGGAGCGACTTTATCTTCAATTTCCTCTAGATCAAAGTCGATGTCATCAAGGTCTTCAATTTCTAATT
CTAGATCTAACTCTTCTTCAGGTGTAACCTTGTCGGCCATTCCCGACCCATTTGCTGTCCACCAGTCATG
CTAGCCATATGGCTGCCGCGCGGCACCAGGCCGCTGCTGTGATGATGATGATGATGGCTGCTGCCCATGG
TA

BhaA-NW

TTGCTCAGCGGTGGCAGCAGCCAACTCAGCTTCCTTTCGGGCTTTGTTAGCAGCCGGATCTCAGTGGTGG
TGGTGGTGGTGCTCGAGTGCGGCCGCAAGCTTGTCGACGGAGCTCGAATTCGGATTACCAGTTAAGAGCC
AGAGGAGCGACTTTATCTTCAATTTCCTCTAGATCAAAGTCGATGTCATCAAGGTCTTCAATTTCTAATT
CTAGATCTAACTCTTCTTCAGGTGTAACCTTGTCGGCCATTCCCGACCCATTTGCTGTCCACCAGTCATG
CTAGCCATATGGCTGCCGCGCGGCACCAGGCCGCTGCTGTGATGATGATGATGATGGCTGCTGCCCATGG
TA

BhaA-SW

TTGCTCAGCGGTGGCAGCAGCCAACTCAGCTTCCTTTCGGGCTTTGTTAGCAGCCGGATCTCAGTGGTGG
TGGTGGTGGTGCTCGAGTGCGGCCGCAAGCTTGTCGACGGAGCTCGAATTCGGATTACCAGCTAAGAGCC
AGAGGAGCGACTTTATCTTCAATTTCCTCTAGATCAAAGTCGATGTCATCAAGGTCTTCAATTTCTAATT
CTAGATCTAACTCTTCTTCAGGTGTAACCTTGTCGGCCATTCCCGACCCATTTGCTGTCCACCAGTCATG
CTAGCCATATGGCTGCCGCGCGGCACCAGGCCGCTGCTGTGATGATGATGATGATGGCTGCTGCCCATGG
TA

BhaA-TW

TTGCTCAGCGGTGGCAGCAGCCAACTCAGCTTCCTTTCGGGCTTTGTTAGCAGCCGGATCTCAGTGGTGG
TGGTGGTGGTGCTCGAGTGCGGCCGCAAGCTTGTCGACGGAGCTCGAATTCGGATTACCAGGTAAGAGCC
AGAGGAGCGACTTTATCTTCAATTTCCTCTAGATCAAAGTCGATGTCATCAAGGTCTTCAATTTCTAATT
CTAGATCTAACTCTTCTTCAGGTGTAACCTTGTCGGCCATTCCCGACCCATTTGCTGTCCACCAGTCATG

CTAGCCATATGGCTGCCGCGCGGCACCAGGCCGCTGCTGTGATGATGATGATGATGGCTGCTGCCCATGG
TA

<u>BhaA-AWG</u>

TCGACGGAGCTCGAATTCGG<u>TTAACCCCAGGCAAGAGCCAGAGGAGCGACTTTATCTTCAATTTCCTCTA
GATCAAAGTCGATGTCATCAAGGTCTTCAATTTCTAATTCTAGATCTAACTCTTCTTCAGGTGTAACCTT
GTCGGCCAT</u>ATCCCGACCCATTTGCTGTC

Table S2. Oligonucleotide and primer sequences for construction of peptide and protein expression plasmids. G_f, gene forward primer; G_r, gene reverse primer; V_f, vector forward primer; V_r, vector reverse primer, *indicates SLIM primers were used to generate mutant

Construct	Primers
pETDuet-BhaA-Ala-Trp	G_f: CCACAGCCAGATGGCCGACAAGG<br>G_r: AATTCGGATCTTACCACGCAAGAGCC<br>V_f: TGCGTGGTAAGATCCGAATTCGAGCT<br>V_r: TGTCGGCCATCTGGCTGTGGTGAT
pETDuet-BhaA-Ala-Trp-Ala*	F_t: ttgcgtgggcgtaaGATCCGAATTCGAGCTCGGCGCG<br>F_s: GGATCttacgcccacgcaagagccagaggagcgactt<br>R_t: CTTAAGCTCGAGCCGCGCGGACGTCC<br>R_s: ctcggtctcctcgctgaaatagaagttaaaggaga
pET28b-His6-BhaA-Ala-Trp-Gly	V_f: CCCGACCCATTTGCTGTCCACCAGTCATGCTAGCCATATGGCTG<br>V_r: TTCGAGCTCCGTCGACAAGCTTGCGGCCGCACT
pET28b-His6-MBP-His6-TEV-10mer	G_f: CTGTACTTCCAATCCGATAAAGTCGCTCCTCTGGC<br>G_r: CCTGAATTGCTTACCACGCAAGAGCCAGAG<br>V_f: GCGTGGTAAGCAATTCAGGGTAGCGGTG<br>V_r: AGGAGCGACTTTATCGGATTGGAAGTACAGGTTCT
pET28b-His6-MBP-His6-TEV-20mer	G_f: CCTGTACTTCCAATCCGACATCGACTTTGATCTAGAGG<br>G_r: CCTGAATTGCTTACCACGCAAGAGCCAGAG<br>V_f: GCGTGGTAAGCAATTCAGGGTAGCGGTG<br>V_r: ATCAAAGTCGATGTCGGATTGGAAGTACAGGTTCTC
pET28b-His6-MBP-His6-TEV-20mer 1-3Ala	G_f:CTGTACTTCCAATCCGCGGCGGCGTTTGATCTAGAGGAAATTGAAGATAAAGTCGCTCCTCTGGCTCTTGCGTGGTAAGCAATTCAGGGTAGC<br>G_r:GCTACCCTGAATTGCTTACCACGCAAGAGCCAGAGGAGCGACTTTATCTTCAATTTCCTCTAGATCAAACGCCGCCGCGGATTGGAAGTACAG<br>V_f:GCTCTTGCGTGGTAAGCAATTCAGGGTAGCGGTGA<br>V_r:ATCAAACGCCGCCGCGGATTGGAAGTACAGGTTCT
pET28b-His6-MBP-His6-TEV-20mer 4-6Ala	G_f:CTGTACTTCCAATCCGACATCGACGCGGCGGCGGAGGAAATTGAAGATAAAGTCGCTCCTCTGGCTCTTGCGTGGTAAGCAATTCAGGGTAG<br>G_r:CTACCCTGAATTGCTTACCACGCAAGAGCCAGAGGAGCGACTTTATCTTCAATTTCCTCCGCCGCCGCGTCGATGTCGGATTGGAAGTACAG<br>V_f:GCTCTTGCGTGGTAAGCAATTCAGGGTAGCGGTGA<br>V_r:CGCCGCGTCGATGTCGGATTGGAAGTACAGGTTCT
pET28b-His6-MBP-His6-TEV-20mer 7-9Ala	G_f:CTGTACTTCCAATCCGACATCGACTTTGATCTAGCGGCGGCGGAAGATAAAGTCGCTCCTCTGGCTCTTGCGTGGTAAGCAATTCAGGGTAG<br>G_r:CTACCCTGAATTGCTTACCACGCAAGAGCCAGAGGAGCGACTTTATCTTCCGCCGCCGCTAGATCAAAGTCGATGTCGGATTGGAAGTACAG<br>V_f:GCTCTTGCGTGGTAAGCAATTCAGGGTAGCGGTGA<br>V_r:ATCAAAGTCGATGTCGGATTGGAAGTACAGGTTCT
pET28b-His6-MBP-His6-TEV-20mer 10-12Ala	G_f:CTGTACTTCCAATCCGACATCGACTTTGATCTAGAGGAAATTGCGGCGGCGGTCGCTCCTCTGGCTCTTGCGTGGTAAGCAATTCAGGGTAG<br>G_r:CTACCCTGAATTGCTTACCACGCAAGAGCCAGAGGAGCGACCGCCGCCGCAATTTCCTCTAGATCAAAGTCGATGTCGGATTGGAAGTACAG<br>V_f:GCTCTTGCGTGGTAAGCAATTCAGGGTAGCGGTGA<br>V_r:ATCAAAGTCGATGTCGGATTGGAAGTACAGGTTCT
pET28b-His6-MBP-His6-TEV-20mer 13-15Ala	G_f:CTGTACTTCCAATCCGACATCGACTTTGATCTAGAGGAAATTGAAGATAAAGCGGCGGCGCTGGCTCTTGCGTGGTAAGCAATTCAGGGTAG<br>G_r:CTACCCTGAATTGCTTACCACGCAAGAGCCAGCGCCGCCGCTTTATCTTCAATTTCCTCTAGATCAAAGTCGATGTCGGATTGGAAGTACAG<br>V_f:GCTCTTGCGTGGTAAGCAATTCAGGGTAGCGGTGA<br>V_r:ATCAAAGTCGATGTCGGATTGGAAGTACAGGTTCT
pET28b-His6-MBP-His6-TEV-20mer 1Ala-ins	G_f: CTGTACTTCCAATCCGACATCGACTTTGATCTAGAGGAAATTGAAGATAAAGTCGCTCCTGCCCTGGCTCTTGCGTGGTAAGCAATTCAGGGTAGC<br>G_r:GCTACCCTGAATTGCTTACCACGCAAGAGCCAGGGCAGGAGCGACTTTATCTTCAATTTCCTCTAGATCAAAGTCGATGTCGGATTGGAAGTACAG<br>V_f: GCGTGGTAAGCAATTCAGGGTAGCGGTG<br>V_r: ATCAAAGTCGATGTCGGATTGGAAGTACAGGTTCTC
pET28b-His6-MBP-His6-TEV-20mer 2Ala-ins	G_f:GCTACCCTGAATTGCTTACCACGCAAGAGCCAGGGCGGCAGGAGCGACTTTATCTTCAATTTCCTCTAGATCAAAGTCGATGTCGGATTGGAAGTACAG<br>G_r:GCTACCCTGAATTGCTTACCACGCAAGAGCCAGGGCGGCAGGAGCGACTTTATCTTCAATTTCCTCTAGATCAAAGTCGATGTCGGATTGGAAGTACAG<br>V_f: GCGTGGTAAGCAATTCAGGGTAGCGGTG<br>V_r: ATCAAAGTCGATGTCGGATTGGAAGTACAGGTTCTC
pET28b-His6-MBP-His6-TEV-20mer 3Ala-ins	G_f:TGTACTTCCAATCCGACATCGACTTTGATCTAGAGGAAATTGAAGATAAAGTCGCTCCTGCCGCCGCCCTGGCTCTTGCGTGGTAAGCAATTCAGGGTAG<br>G_r:CTACCCTGAATTGCTTACCACGCAAGAGCCAGGGCGGCGGCAGGAGCGACTTTATCTTCAATTTCCTCTAGATCAAAGTCGATGTCGGATTGGAAGTACA<br>V_f: GCGTGGTAAGCAATTCAGGGTAGCGGTG<br>V_r: ATCAAAGTCGATGTCGGATTGGAAGTACAGGTTCTC
pET28b-His6-MBP-His6-TEV-20mer 4Ala-ins	G_f: TACTTCCAATCCGACATCGACTTTGATCTAGAGGAAATTGAAGATAAAGTCGCTCCTGCCGCCGCCGCCCTGGCTCTTGCGTGGTAAGCAATTCAGGGT<br>G_r: ACCCTGAATTGCTTACCACGCAAGAGCCAGGGCGGCGGCGGCAGGAGCGACTTTATCTTCAATTTCCTCTAGATCAAAGTCGATGTCGGATTGGAAGTA<br>V_f: GCGTGGTAAGCAATTCAGGGTAGCGGTG<br>V_r: ATCAAAGTCGATGTCGGATTGGAAGTACAGGTTCTC
pET28b-His6-MBP-His6-TEV-20mer 5Ala-ins	G_f:ACTTCCAATCCGACATCGACTTTGATCTAGAGGAAATTGAAGATAAAGTCGCTCCTGCCGCCGCCGCCGCCCTGGCTCTTGCGTGGTAAGCAATTCAGGG<br>G_r:CCCTGAATTGCTTACCACGCAAGAGCCAGGGCGGCGGCGGCGGCAGGAGCGACTTTATCTTCAATTTCCTCTAGATCAAAGTCGATGTCGGATTGGAAGT<br>V_f: GCGTGGTAAGCAATTCAGGGTAGCGGTG<br>V_r: ATCAAAGTCGATGTCGGATTGGAAGTACAGGTTCTC
pET28b-His6-His6-TEV-	G_f: CAAATGGGTCGGGATATGGCCGACAAGGTTACACC<br>G_r: CGGAGCTCGAATTCGGTTACCAATCAAGAGCCAGAGG<br>V_f: GGCTCTTGATTGGTAACCGAATTCGAGCTCCGTCGAC

20mer A19D	V_r: AACCTTGTCGGCCATATCCCGACCCATTTGCTGTC
pET28b-His6-His6-TEV-20mer A19K	G_f: CAAATGGGTCGGGATATGGCCGACAAGGTTACACC<br>G_r: GTCGACGGAGCTCGAATTCGGTTACCATTTAAGAGCCAGAG<br>V_f: TGGCTCTTAAATGGTAACCGAATTCGAGCTCCGTCGAC<br>V_r: AACCTTGTCGGCCATATCCCGACCCATTTGCTGTC
pET28b-His6-His6-TEV-20mer A19N	G_f: CAAATGGGTCGGGATATGGCCGACAAGGTTACACC<br>G_r: ACGGAGCTCGAATTCGGTTACCAGTTAAGAGCCAGAGG<br>V_f: TGGCTCTTAACTGGTAACCGAATTCGAGCTCCGTCGAC<br>V_r: AACCTTGTCGGCCATATCCCGACCCATTTGCTGTC
pET28b-His6-His6-TEV-20mer A19S	G_f: CAAATGGGTCGGGATATGGCCGACAAGGTTACACC<br>G_r: AGCTCGAATTCGGTTACCAGCTAAGAGCCAGAGGAGC<br>V_f: GCTCTTAGCTGGTAACCGAATTCGAGCTCCGTCGAC<br>V_r: AACCTTGTCGGCCATATCCCGACCCATTTGCTGTC
pET28b-His6-His6-TEV-D20mer A19T	G_f: CAAATGGGTCGGGATATGGCCGACAAGGTTACACC<br>G_r: GGAGCTCGAATTCGGTTACCAGGTAAGAGCCAGAGGAGC<br>V_f: GCTCTTACCTGGTAACCGAATTCGAGCTCCGTCGAC<br>V_r: AACCTTGTCGGCCATATCCCGACCCATTTGCTGTC
pET28b-His6-His6-TEV-20mer A19F	G_f: CAAATGGGTCGGGATATGGCCGACAAGGTTACACC<br>G_r: ACGGAGCTCGAATTCGGTTACCAAAAAAGAGCCAGAGG<br>V_f: TGGCTCTTTTTTGGTAACCGAATTCGAGCTCCGTCGAC<br>V_r: AACCTTGTCGGCCATATCCCGACCCATTTGCTGTC
pET28b-His6-His6-TEV-20mer A19G	G_f: CAAATGGGTCGGGATATGGCCGACAAGGTTACACC<br>G_r: GGAGCTCGAATTCGGTTACCAGCCAAGAGCCAGAG<br>V_f: CTCTTGGCTGGTAACCGAATTCGAGCTCCGTCGAC<br>V_r: AACCTTGTCGGCCATATCCCGACCCATTTGCTGTC
pET28b-His6-His6-TEV-20mer A19P	G_f: CAAATGGGTCGGGATATGGCCGACAAGGTTACACC<br>G_r: AGCTCGAATTCGGTTACCACGGAAGAGCCAGAGGAG<br>V_f: CTCTTCCGTGGTAACCGAATTCGAGCTCCGTCGAC<br>V_r: AACCTTGTCGGCCATATCCCGACCCATTTGCTGTC
pET28b-His6-His6-TEV-20mer A19V	G_f: CAAATGGGTCGGGATATGGCCGACAAGGTTACACC<br>G_r: GGAGCTCGAATTCGGTTACCACACAAGAGCCAGAGG<br>V_f: GCTCTTGTGTGGTAACCGAATTCGAGCTCCGTCGAC<br>V_r: AACCTTGTCGGCCATATCCCGACCCATTTGCTGTC
pET28b-His6-His6-TEV-20mer A19W	G_f: CAAATGGGTCGGGATATGGCCGACAAGGTTACACC<br>G_r: GGAGCTCGAATTCGGTTACCACCAAAGAGCCAGAGG<br>V_f: GCTCTTTGGTGGTAACCGAATTCGAGCTCCGTCGAC<br>V_r: AACCTTGTCGGCCATATCCCGACCCATTTGCTGTC
pET28b-His6-MBP-His6-TEV-19mer del-A19	G_f: CTGTACTTCCAATCCGACATCGACTTTGATCTAGAGGAAATTGAAGATAAAGTCGCTCCTCTGGCTCTTTGGTAAGCAATTCAGGGTAGC<br>G_r: GCTACCCTGAATTGCTTACCAAAGAGCCAGAGGAGCGACTTTATCTTCAATTTCCTCTAGATCAAAGTCGATGTCGGATTGGAAGTACAG<br>V_f: CTGGCTCTTTGGTAAGCAATTCAGGGTAGC<br>V_r: ATCAAAGTCGATGTCGGATTGGAAGTACAGGTTCTC
pET28b-His6-MBP-His6-TEV-18mer del-L18, del-A19	G_f: CTGTACTTCCAATCCGACATCGACTTTGATCTAGAGGAAATTGAAGATAAAGTCGCTCCTCTGGCTTGGTAAGCAATTCAGGGTAGC<br>G_r: CTACCCTGAATTGCTTACCAAGCCAGAGGAGCGACTTTATCTTCAATTTCCTCTAGATCAAAGTCGATGTCGGATTGGAAGTACAG<br>V_f: CCTCTGGCTTGGTAAGCAATTCAGGGTAGC<br>V_r: ATCAAAGTCGATGTCGGATTGGAAGTACAGGTTCTC
pET28b-His6-MBP-His6-TEV-17mer del-A17, del-L18, del-A19	G_f: CTGTACTTCCAATCCGACATCGACTTTGATCTAGAGGAAATTGAAGATAAAGTCGCTCCTCTGTGGTAAGCAATTCAGGGTAGC<br>G_r: GCTACCCTGAATTGCTTACCACAGAGGAGCGACTTTATCTTCAATTTCCTCTAGATCAAAGTCGATGTCGGATTGGAAGTACAG<br>V_f: GCTCCTCTGTGGTAAGCAATTCAGGGTAGC<br>V_r: ATCAAAGTCGATGTCGGATTGGAAGTACAGGTTCTC
pET28b-His6-MBP-His6-TEV-16mer del -L16, del-A17, del-L18, del-A19	G_f: CTGTACTTCCAATCCATGGACATCGACTTTGATCTAGAGGAAATTGAAGATAAAGTCGCTCCTTGGTAAGCAATTCAGGGTAGC<br>G_r: GCTACCCTGAATTGCTTACCAAGGAGCGACTTTATCTTCAATTTCCTCTAGATCAAAGTCGATGTCCATGGATTGGAAGTACAG<br>V_f:GTCGCTCCTTGGTAAGCAATTCAGGGTAGC<br>V_r:ATCAAAGTCGATGTCGGATTGGAAGTACAGGTTCTC
pET28b-His6-MBP-His6-TEV-20mer L18A	G_f: CTGTACTTCCAATCCGACATCGACTTTGATCTAGAGGAAATTGAAGATAAAGTCGCTCCTCTGGCTGCTGCTTGGTAAGCAATTCAGGGTAGC<br>G_r: GCTACCCTGAATTGCTTACCAAGCAGCAGCCAGAGGAGCGACTTTATCTTCAATTTCCTCTAGATCAAAGTCGATGTCGGATTGGAAGTACAG<br>V_f:GCTGCTGCTTGGTAAGCAATTCAGGGTAGC<br>V_r:ATCAAAGTCGATGTCGGATTGGAAGTACAGGTTCTC
pET28b-His6-MBP-	G_f: CTGTACTTCCAATCCGACATCGACTTTGATCTAGAGGAAATTGAAGATAAAGTCGCTCCTCTGGCTGAGGCTTGGTAAGCAATTCAGGGTAGC<br>G_r: GCTACCCTGAATTGCTTACCAAGCCTCAGCCAGAGGAGCGACTTTATCTTCAATTTCCTCTAGATCAAAGTCGATGTCGGATTGGAAGTACAG

His6-TEV-<br>20mer<br>L18E	V_f:GCTGAGGCTTGGTAAGCAATTCAGGGTAGC<br>V_r:ATCAAAGTCGATGTCGGATTGGAAGTACAGGTTCTC
pET28b-<br>His6-MBP-<br>His6-TEV-<br>20mer<br>L18F	G_f: CTGTACTTCCAATCCGACATCGACTTTGATCTAGAGGAAATTGAAGATAAAGTCGCTCCTCTGGCTTTTGCTTGGTAAGCAATTCAGGGTAGC<br>G_r: GCTACCCTGAATTGCTTACCAAGCAAAAGCCAGAGGAGCGACTTTATCTTCAATTTCCTCTAGATCAAAGTCGATGTCGGATTGGAAGTACAG<br>V_f:GCTTTTGCTTGGTAAGCAATTCAGGGTAGC<br>V_r:ATCAAAGTCGATGTCGGATTGGAAGTACAGGTTCTC
pET28b-<br>His6-MBP-<br>His6-TEV-<br>20mer<br>L19K	G_f: CTGTACTTCCAATCCGACATCGACTTTGATCTAGAGGAAATTGAAGATAAAGTCGCTCCTCTGGCTAAAGCTTGGTAAGCAATTCAGGGTAGC<br>G_r: GCTACCCTGAATTGCTTACCAAGCTTTAGCCAGAGGAGCGACTTTATCTTCAATTTCCTCTAGATCAAAGTCGATGTCGGATTGGAAGTACAG<br>V_f:GCTAAAGCTTGGTAAGCAATTCAGGGTAGC<br>V_r:ATCAAAGTCGATGTCGGATTGGAAGTACAGGTTCTC
pET28b-<br>His6-MBP-<br>His6-TEV-<br>20mer<br>P15A	G_f: CTGTACTTCCAATCCGACATCGACTTTGATCTAGAGGAAATTGAAGATAAAGTCGCTGCTCTGGCTCTTGCTTGGTAAGCAATTCAGGGTAGC<br>G_r: CAGAGCAGCGACTTTATCTTCAATTTCCTCTAGATCAAAGTCGATGTCGGATTGGAAGTACAG<br>V_f:GCTCTTGCTTGGTAAGCAATTCAGGGTAGC<br>V_r:ATCAAAGTCGATGTCGGATTGGAAGTACAGGTTCTC

Table S3. Calculated and observed masses of ions in Figures 3 and 4 of main text. * denotes $[M + 3 O]^+$ product.

Figure 3		
**Species**	**Calculated m/z**	**Observed m/z**
WT 20mer	2389.2 Da	2389.0 Da
WT 20mer*	2437.1 Da	2436.9 Da
P15A	2363.3 Da	2363.4 Da
P15A*	2411.3 Da	2411.5 Da
10-12Ala	2230.1 Da	2230.3 Da
13-15Ala	2335.1 Da	2335.8 Da
13-15Ala*	2383.1 Da	2383.7 Da

Figure 3		
**Species**	**Calculated m/z**	**Observed m/z**
WT 20mer	2389.2 Da	2389.0 Da
WT 20mer*	2437.1 Da	2436.9 Da
1Ala-ins	2460.2 Da	2459.5 Da
1Ala-ins*	2508.2 Da	2507.6 Da
2Ala-ins	2531.3 Da	2530.5 Da
2Ala-ins*	2579.3 Da	2578.5 Da
3Ala-ins	2602.3 Da	2601.4 Da
3Ala-ins*	2650.3 Da	2649.4 Da
4Ala-ins	2673.3 Da	2672.9 Da
4Ala-ins*	2721.3 Da	2720.9 Da
5Ala-ins	2744.4 Da	2743.5 Da
5Ala-ins*	2792.4 Da	2791.5 Da

Table S4. AlphaFold pLDDT values for BhaA-Ala-Trp and AmmA*-Trp for each residue within each sequence. Average pLDDT for BhaA-Ala-Trp = 40.14. Average pLDDT for AmmA*-Trp = 32.25.

BhaA-Ala-Trp		
**Position**	**Residue**	**pLDDT**
1	Met	34.7
2	Ala	35.8
3	Asp	37.5
4	Lys	38.3
5	Val	46.1
6	Thr	48.9
7	Pro	48.9
8	Glu	46.6
9	Glu	49.1
10	Glu	46.0
11	Leu	45.0
12	Asp	45.7
13	Leu	43.3
14	Glu	42.6
15	Leu	39.9
16	Glu	41.9
17	Ile	39.0
18	Glu	39.9
19	Asp	39.6
20	Leu	41.4
21	Asp	38.5
22	Asp	38.3
23	Ile	39.6
24	Asp	38.6
25	Phe	40.9
26	Asp	38.3
27	Leu	35.7
28	Glu	38.0
29	Glu	38.7
30	Ile	40.0
31	Glu	37.0
32	Asp	40.0
33	Lys	39.5
34	Val	41.9
35	Ala	38.7
36	Pro	38.3
37	Leu	36.7
38	Ala	36.6
39	Leu	34.6
40	Ala	34.0
41	Trp	31.3

AmmA*-Trp		
**Position**	**Residue**	**pLDDT**
1	Met	23.9
2	Ser	22.7
3	Glu	23.2
4	Thr	25.7
5	Gln	22.8
6	Val	22.1
7	Thr	22.7
8	Glu	23.8
9	Thr	24.6
10	Asp	28.5
11	Asn	26.4
12	Pro	24.4
13	Ala	26.9
14	Glu	25.2
15	Glu	24.3
16	Pro	26.4
17	Ala	29.7
18	Glu	31.7
19	Ile	29.9
20	Ala	30.9
21	Ala	31.5
22	Glu	33.0
23	Ser	31.7
24	Asp	34.8
25	Asp	38.6
26	Leu	37.6
27	Ala	36.4
28	Asp	39.2
29	Leu	37.6
30	Asp	36.1
31	Asp	39.3
32	Ile	39.0
33	Glu	40.6
34	Phe	39.4
35	Asp	43.5
36	Leu	37.1
37	Asp	38.5
38	Glu	37.6
39	Val	36.2
40	Glu	37.4
41	Ser	34.0
42	Lys	38.1
43	Ile	35.2
44	Ala	35.9
45	Pro	34.7
46	Leu	37.5
47	Ala	34.4
48	Leu	37.0
49	Ala	35.4
50	Trp	30.1

References

(1) Ting, C. P., Funk, M. A., Halaby, S. L., Zhang, Z., Gonen, T., and van der Donk, W. A. (2019) Use of a scaffold peptide in the biosynthesis of amino acid-derived natural products, *Science 365*, 280-284.

(2) Daniels, P. N., Lee, H., Splain, R. A., Ting, C. P., Zhu, L., Zhao, X., Moore, B. S., and van der Donk, W. A. (2022) A biosynthetic pathway to aromatic amines that uses glycyl-tRNA as nitrogen donor, *Nat. Chem. 14*, 71-77.

(3) Pettersen, E. F., Goddard, T. D., Huang, C. C., Meng, E. C., Couch, G. S., Croll, T. I., Morris, J. H., and Ferrin, T. E. (2021) UCSF ChimeraX: Structure visualization for researchers, educators, and developers, *Protein Sci. 30*, 70-82.
